# PD-1 derived CA-170 is an oral immune checkpoint inhibitor that exhibits preclinical anti-tumor efficacy

**DOI:** 10.1038/s42003-021-02191-1

**Published:** 2021-06-08

**Authors:** Pottayil G. Sasikumar, Naremaddepalli S. Sudarshan, Srinivas Adurthi, Raghuveer K. Ramachandra, Dodderi S. Samiulla, Anirudha Lakshminarasimhan, Anuradha Ramanathan, Talapaneni Chandrasekhar, Amit A. Dhudashiya, Sumalatha R. Talapati, Nagesh Gowda, Sreenivasulareddy Palakolanu, Jiju Mani, Bandi Srinivasrao, David Joseph, Nigam Kumar, Rashmi Nair, Hanudatta S. Atreya, Nagaraj Gowda, Murali Ramachandra

**Affiliations:** 1 Aurigene Discovery Technologies Limited, Bangalore, Karnataka India; 2 NMR Research Centre, Indian Institute of Science, Bangalore, Karnataka India

**Keywords:** Small molecules, Drug discovery

## Abstract

Small molecule immune checkpoint inhibitors targeting PD-1 and other pathways may offer advantages including ease of dosing, ability to manage immune-related adverse events (irAEs) due to their shorter pharmacokinetic exposure and opportunity to target more than one pathway for improving efficacy. Here we describe the identification and characterization of CA-170, an amino acid inspired small molecule inhibitor of PD-L1 and VISTA derived from the interface of PD-1 and PD-L1. CA-170 exhibited potent rescue of proliferation and effector functions of T cells inhibited by PD-L1/L2 and VISTA with selectivity over other immune checkpoint proteins as well as a broad panel of receptors and enzymes. Observed blocking of PD-L1 signaling and binding to PD-L1 in the cellular context without preventing the assembly of PD-1:PD-L1 complex support the formation of a defective ternary complex as the mechanism of action of CA-170. Oral administration of CA-170 resulted in increased proliferation and activation of T cells in the tumor, and significant anti-tumor efficacy in a number of immunocompetent mouse tumor models either as a single agent or in combination with approved therapeutics. These results prompted the advancement of CA-170 to human clinical trials.

## Abstract (web summary)

Sasikumar et al. describe the identification and characterization of CA-170, a small molecule inhibitor of PD-L1 and VISTA. They find that CA-170 activates T cells and exhibits anti-tumor efficacy in mouse models. This study highlights the potential of CA-170, which has advanced to human clinical trials, as an anti-cancer drug.

## Introduction

The immune system is a significant player in the battle against cancer to regulate and eliminate malignant cells as evidenced by the well-accepted use of inhibitors targeting immune checkpoint pathways and adoptive cellular therapy^1^. Discovery, development, and clinical practice of inhibitory antibodies targeting CTLA-4 and PD-1 pathways are acknowledged with the well-deserved nobel prize in Physiology or Medicine in 2018^2^. Apart from these pathways, there are other checkpoint pathways such as VISTA expressed in the tumor microenvironment (TME) that play a role in dampening the anti-tumor immune response and come in the way of achieving effective therapy^3–5^. Interestingly, these immune checkpoint pathways in TME are non-redundant thus providing an opportunity for simultaneous targeting of more than one checkpoint protein towards overcoming the immune tolerance in the tumor.

Antibodies are widely used to antagonize the immune checkpoint pathways^6^. While these antibody-based therapies show impressive clinical activity, they suffer from a few shortcomings. One of the major limitations of approved antibodies is their response only in a minority of patients partly due to the compensatory inhibitory mechanism by upregulation of alternative immune checkpoints such as TIM-3 and VISTA^7,8^. Other limitations include immune-related adverse events (irAEs) due to the breaking of immune self-tolerance, tissue accessibility and need to administer by intravenous injection^9–11^. Deficiencies of antibody-based inhibitors underscore the need for alternate approaches including small molecule agents that are amenable for oral dosing, capable of targeting more than one immune checkpoint protein for greater clinical response rate and allow better management of irAEs due to a shorter pharmacokinetic (PK) profile and suitable for combination with other approved chemotherapeutic drugs.

Development of small-molecule immune checkpoint inhibitors has not kept up with antibody drug discovery likely due to inherent challenges in identifying a small molecule capable of preventing protein–protein interactions. Challenges in targeting immune checkpoint ligand–receptor interaction include the need to affect relatively large and amorphous interfaces, rather than small and well-defined pockets. Transient receptor–ligand interaction with a relative weak binding affinity and possibility of allosteric binding and/or partial blocking of the interaction leading to functional antagonism offer the possibility of identifying small molecule agents. Interestingly, in comparison to the most of reported antibodies and small molecule agents, the reports of potent and pharmacologically active anti-PD-1 antibodies despite their inability to block the interaction of PD-1 with PD-L1^12,13^ also support the possibility of identifying agents with a distinct mechanism of action. The ligand–receptor interaction interface comprises of short continuous peptide epitopes consisting of linear sequences, secondary structural epitopes such as a single face of an α-helix binding to a hydrophobic groove of complementary residues and the tertiary structure of epitopes wherein the binding interface is not continuous requiring multiple sites to form the interface. The interfaces of tertiary epitopes are more prevalent and dynamic, thus representing an excellent opportunity to target with small molecule agents^14^.

One of the widely used approaches to identify small molecule agents is to screen a large library of compounds in a binding assay comprising of extracellular domains of receptors and ligands typically expressed in a heterologous system such as the bacterial system. This approach and subsequent modifications have resulted in the identification of small molecules targeting PD-1/PD-L1 interaction by BMS and other groups^15–18^. In parallel to the published approaches, we adapted a different method in which a high-affinity peptide from the receptor–ligand interface sequence is first identified followed by conversion of the minimal pharmacophore in the peptide in to an amino acid-inspired heterocyclic compound. The first step in our approach resulted in a peptide inhibitor for PD-1 pathway as reported in 2011 and published later^19,20^. In our approach, rational design based on the interface sequence substituted for the large compound library screening, while the use of a functional assay using the native system to assess the compound replaced the binding assay using recombinant proteins.

Here we report the discovery of an orally available small molecule immune checkpoint inhibitor, CA-170, dually targeting PD-L1 and VISTA pathways. CA-170 exhibited potent functional activity in rescuing T-cell proliferation and effector functions, while showing selectivity against other immune checkpoint proteins as well as in a broad panel of receptors and enzymes. CA-170 showed binding to PD-L1 in the cellular context without preventing the assembly of PD-1:PD-L1 complex supporting the formation of a defective ternary complex as the mode of action. In multiple preclinical models, CA-170 showed efficacy in inhibition of tumor growth at well-tolerated doses. This is the first small molecule oral immune checkpoint inhibitor that progressed to the clinic (ClinicalTrials.gov Identifier: NCT02812875)^21^.

## Results

### Discovery of an orally bioavailable PD-L1 inhibitor

Major checkpoint receptors belong to B7 family and the members of this family and their known ligands are part of the immunoglobulin (IgG) superfamily comprising of shared structural features with IgG and possessing an IgG domain or fold^22^. The checkpoint ligand–receptor interactions are classic examples of protein–protein interactions for which we reasoned that the peptide fragments from the interaction interface would be ideal starting points for the inhibitor design. A series of peptides spanning the loop and strand sequences of PD-1 from the interface of PD-1 and PD-L1 was synthesized. These peptides were analyzed for their ability to rescue T cells from the inhibitory activity of PD-L1 in a splenocytes/PBMC-based functional assay. Among these peptides, a peptide spanning the BC loop was found to be active^20^. Interestingly, the functional activity was also observed when this peptide was truncated to a three amino acid peptide containing amino acids, SNT (PM-116). As expected for unmodified peptides, both these peptides were highly unstable in the plasma (Table 1). Head to tail cyclization of the linear analogues of SNTSESF (PM-823) was stable in plasma but was not orally bioavailable.

**Table 1:** Evolution of CA-170: profiles of hit and lead compounds.

#	Structure	Class of compound	Rescue of proliferation EC_50_ nM	Plasma stability (% remaining at 4 h)			Mice PK parameters				
				Mice	Rat	Human	Clearance (mL/hr/kg)	AUCi iv(0-α) (hr*ng/mL)	AUC po (0-α) (hr*ng/mL)	*C*max (ng/ml)	% *F*
NP-01		Linear peptide	81	<30	<30	<30	Not analyzed due to low plasma stability of the compound				
PM-823		Head to tail cyclic peptide	55	>95	>95	>95	1376	2185	495	554.21	6.8
PM-116		Linear peptide	6	<30	<30	<30	Not analyzed due to low plasma stability of the compound				
PM-13		Linear peptidomimetic	21 ± 2.8	37	30	37	10697	344	653	342	16.2
PM-105		PM-13 Retro-inverso	33 ± 2.8	62	64	67	1011	3000	2600	2908	15.5
PM-209		Cyclic version of PM-13	40.3 ± 10.4	>95	91	>95	743	4032	5502	3730	13.6
PM-219		PM-209 Retro-inverso	32.6 ± 10.5	>95	90	82	327	9535	11553	6437	13
CA-170		Amino acid fused heterocycles	15.4 ± 1.3	>95	>95	>95	723	4205	19574	1723	50

Functional activity was tested in a splenocyte-based assay by measuring the ability of the test peptides to rescue PD-L1 mediated inhibition of proliferation. For initial hit compounds such as NP-01, PM-823, and PM-116 data is presented as EC_50_ value for rescue of proliferation from a set of triplicate values (*n* = 1). For all lead compounds PM-13, PM-105, PM-209, PM-219, and CA-170 data is represented as mean ± S.D of two independent studies. Mice, rat, and human plasma stability values were represented as mean of duplicate of percent parent compound remaining at 4 h, tested at 10 μM. Mice PK parameters including oral bioavailability were determined from intravenous and oral route of administration of compounds at 1 mg kg^−1^ and 30 mg kg^−1^, respectively.

Towards stabilizing the peptide SNT to achieve oral bioavailability, a series of modifications were incorporated. Representative structures that retained the activity along with their metabolic stability and PK profile are summarized in Table 1. Linear peptidomimetics were synthesized in which two amide bonds in SNT were replaced by non-peptidic bonds. Among the various bonds analyzed, the diacylhydrazine bond between S and N, and urea bond between N and T resulted in a better functional activity. However, these linear peptidomimetics and their cyclic versions also showed very low oral bioavailability. This prompted us to switch the strategy from linear peptidomimetics to amino acid-derived heterocycles. The heterocyclic compounds were obtained by fusing the amino acids resulting in a 1,2,4-oxadizole template which could potentially anchor desired amino acid side chains to interact with residues on PD-L1. Heterocycle moiety was further extended by introducing the remaining amino acid as in the peptide pharmacophore by introducing groups such as urea linkages to connect to the oxadizole template. As shown in Table 1, these changes led to the identification of CA-170 which showed improved metabolic stability and oral bioavailability while retaining functional activity.

### CA-170 selectively inhibits PD-1 and VISTA pathways

**Fig. 1:**
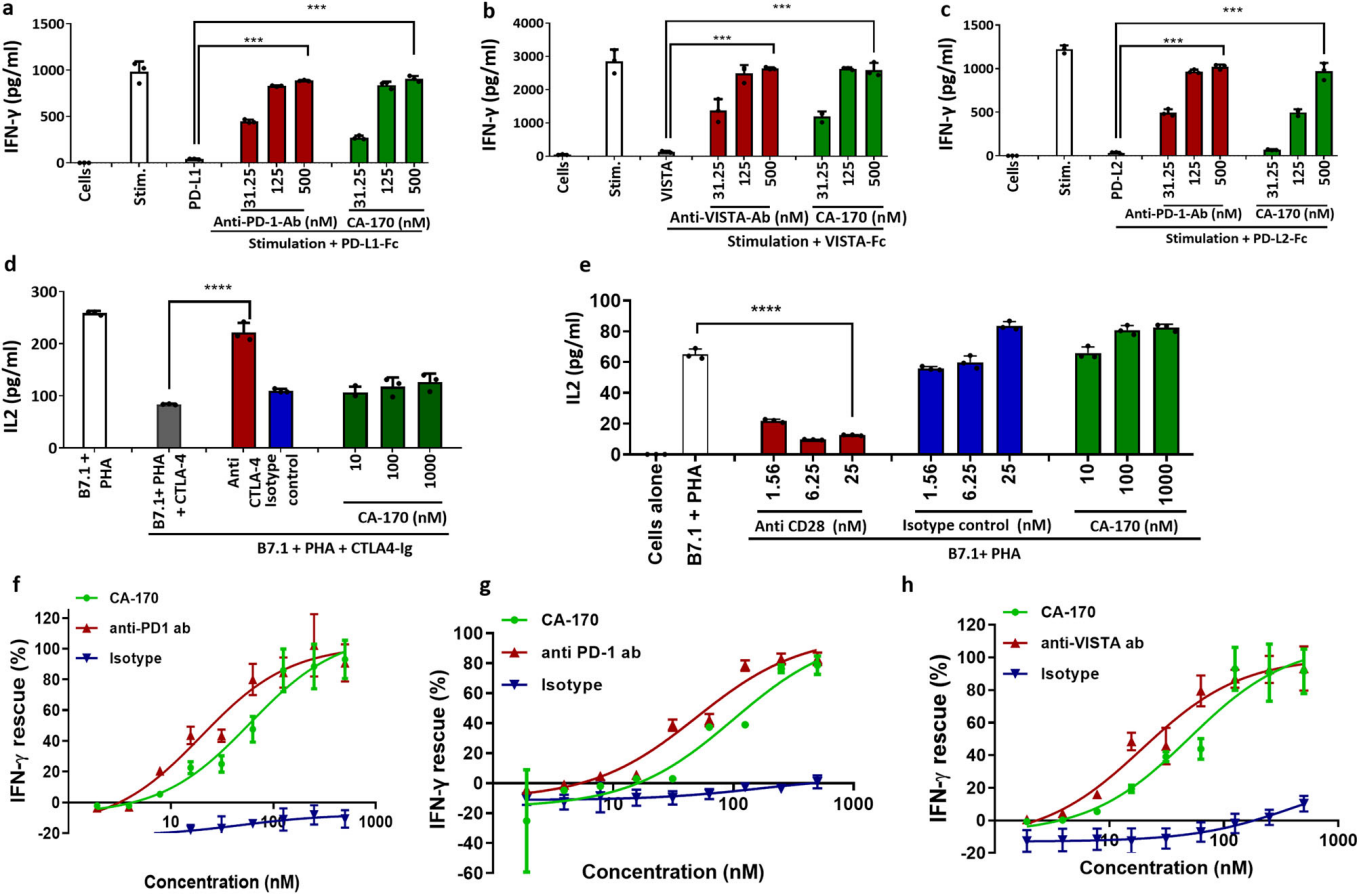
CA-170 is selective for PD-L1, PD-L2, and VISTA among the tested B7 family checkpoint pathways. **a** CA-170 rescued IFNγ release by human PBMCs when stimulated with anti-CD3 and anti-CD28 antibodies, and inhibited by recombinant PD-L1. **b** CA-170 rescued IFNγ release by human PBMCs when stimulated with anti-CD3 and anti-CD28 antibodies, and inhibited by recombinant VISTA. **c** CA-170 rescued IFNγ release by human PBMCs when stimulated with anti-CD3 and anti-CD28 antibodies, and inhibited by recombinant PD-L2. **d** CA-170 did not rescue the release of IL-2 in Jurkat cells stimulated with PHA in the presence of exogenous human B7-1 and inhibited by recombinant hCTLA-4. **e** CA-170 did not inhibit IL-2 secretion in Jurkat cells stimulated with PHA in the presence of exogenous human B7-1. **f** Dose response of rescue of IFNγ release by human PBMCs stimulated with anti-CD3 and anti-CD28 antibodies and inhibited by PD-L1 upon treatment with CA-170, anti-PD-1 antibody or isotype control. **g** Dose response of rescue of IFNγ release by human PBMCs stimulated with anti-CD3 and anti-CD28 antibodies and inhibited by PD-L2 upon treatment with CA-170, anti-PD-1 antibody, or isotype control. **h** Dose response of rescue of IFNγ release by human PBMCs stimulated with anti-CD3 and anti-CD28 antibodies and inhibited by VISTA upon treatment with CA-170, anti-VISTA antibody, or isotype control. One representative data is presented out of three (**a**–**g**) or four (**h**) independent experiments. Each bar/data point in the figures denotes mean ± SD from the three replicates ****p < 0.0001 as calculated by one-way ANOVA (Dunnett’s multiple comparisons test).

CA-170 was evaluated for its activity in functional assays for other key immune pathways in the B7 ligand/receptor family such as CTLA4, PD-L2, VISTA, and immune stimulatory B7-1 (Fig. 1a–e). In all these assays, when stimulated the respective antibodies showed response as expected, while CA-170 was able to only rescue IFNγ release from PBMC inhibited by recombinant PD-L1, PD-L2, and VISTA (Fig. 1a–c), and not IL-2 secretion from Jurkat cells inhibited by CTLA4-Ig (Fig. 1d). Since B7-1 is a closely related ligand that is immunostimulatory^23^, we also evaluated the impact of CA-170 on B7-1-mediated activation. As shown in Fig. 1e, an antibody targeting CD-28 blocked the B7-1-mediated stimulation. CA-170 did not inhibit the IL-2 levels in B7.1+ PHA stimulated cells similar to isotype control, which indicates that CA-170 does not inhibit the B7.1-CD28 co-stimulatory pathway. Dose–response studies showed that CA-170 rescues IFNγ release inhibited by PD-L1, PD-L2, and VISTA with potencies comparable to commercially available antibodies (Fig. 1f–h). The average EC_50_ data from three independent experiments for rescue of proliferation and IFNγ release are tabulated in Supplementary Table 2. When similar assays were done using mouse splenocyte and non-human primate PBMCs in the presence of PD-L1, CA-170 also showed potent activity in rescuing the splenocytes/PBMC proliferation and IFN-γ release inhibited by PD-L1 (Supplementary Table 3), indicating its activity in these preclinical species.

**Fig. 2:**
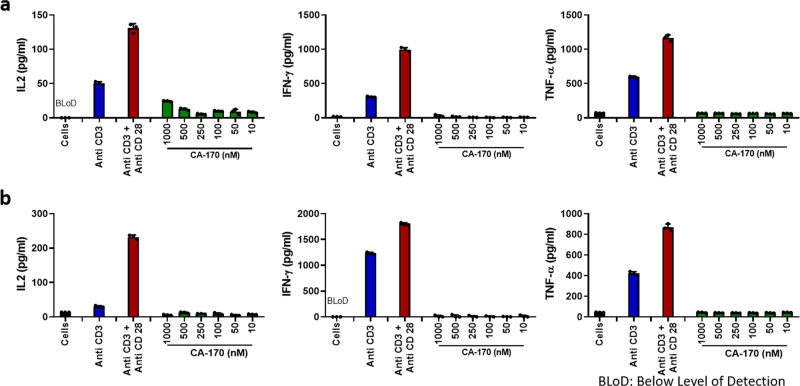
In vitro cytokine induction assay. **a** Human PBMCs from healthy donors were pre-cultured at high density (1 × 10^7^ cells/ml) for 48 h to increase cell–cell contacts and immune scanning. The cells were then harvested and treated with anti-CD3 or anti-CD3 + anti-CD28 antibodies as positive controls, and CA-170 (10 nM to 1000 nM). Release of IL-2, IFN-γ, and TNF-α was measured in the culture supernatants at 48 h. Two donors were used in this study (one male and one female); there was no gender difference observed in the cytokine response in this study. Data presented is representative data from a male donor (BLoD Below Limit of Detection). **b** Cytokine levels determined in PBMCs from the same donor with similar experimental conditions but cultured at a normal density of 1 × 10^5^ cells/well for 48 h for comparison. For **a**–**b** each bar in the figure denotes mean ± SD from the three replicates.

In view of CA-170’s ability to neutralize PD-L1/L2 and VISTA, it was also important to ascertain that this compound does not by itself induce cytokine secretion as reported earlier for an antibody targeting CD28^24^. In the widely used in vitro cytokine induction assay using human PBMCs, CA-170 did not induce significant levels of cytokines in both normal culture conditions or high-density culture conditions, while an antibody against CD28 induced as expected (Fig. 2a, b; Supplementary Table 4).

### CA-170 is selective over a broad panel of receptors and enzymes

To further confirm the selectivity, CA-170 was profiled against a broad panel of receptors and enzymes (80-panel, CEREP profiling assay), and results indicated minimal or no interaction with any of these proteins (Supplementary Table 5).

### CA-170 binds to PD-L1 without preventing the PD-1:PD-L1 complex formation

In view of the functional rescue from the inhibitory activity of PD-L1 in PBMC-based assays by CA-170, we sought to determine the interaction of CA-170 with PD-L1. Consistent with published findings^25–27^, a definitive binding could not be established in widely used techniques such as SPR and solution NMR using recombinant proteins containing extracellular domains. To recapitulate the interaction as in the native environment, we utilized NMR spectroscopy using PD-L1 overexpressing CHO-K1 cells. Cell-based NMR spectroscopy is an ideal tool for studying the binding of macromolecules with a ligand under the physiological environment to obtain biologically relevant structural and functional information^28,29^. This is achieved by monitoring the difference in the nuclear-spin relaxation or transverse relaxation (*T*_2_) rates of a small molecule ligand in the presence and the absence of the macromolecular targets such as cells^30^. Small, rapidly tumbling molecules have much slower relaxation rates (*T*_2_) than slowly reorienting cells and therefore the *T*_2_ of small molecules is generally large compared to the *T*_2_ of cells. Because of the dynamic nature of the interaction, the intensity of the ligand signals in the presence of cells is a weighted average of the free and the bound ligands. The ligands bound to cells attain the NMR properties of cells and show lower *T*_2_ values^30^. During the analysis, a gradual increase in the *T*_2_ value of the small molecule ligand is observed as its concentration is increased because of the increase in free ligand concentration.

**Fig. 3:**
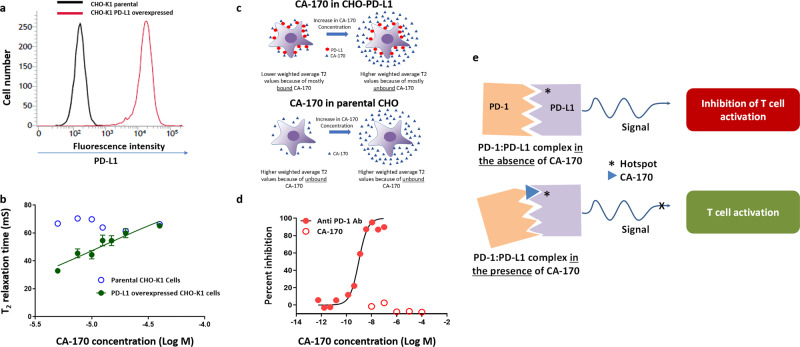
CA-170 specifically binds to PD-L1 overexpressing cells, but fails to prevent PD-1:PD-L1 complex formation. **a** Full-length human PD-L1 was overexpressed in CHO-K1 cells by lentiviral transduction. Single clones of PD-L1 overexpressing cells were isolated by limiting dilution method. The expression of PD-L1 on the parental and PD-L1 overexpressed single clones was determined by staining with anti-human PD-L1–PeCy7 antibody followed by flow cytometry. **b** The *T*_2_ values obtained following the recording of ^13^C-^1^H HSQC spectra with delay and without the delay from parental and PD-L1 overexpressing CHO-K1 cells treated with indicated concentrations of CA-170 are shown from three independent trials. **c** Schematic representation of the data observed from the NMR study from PD-L1 overexpressed cells and parental cells. **d** Anti-PD1 antibody or CA-170 were tested at the indicated concentrations in a TR-FRET-based PD-1:PD-L1 complex formation assay. The results are represented as mean of duplicates. **e** A schematic depicting the proposed mechanism-of-action for CA-170 in blocking PD-L1 action. In conjunction with the blocking of PD-L1 signaling as observed in functional assays and binding to PD-L1 in the cellular context without preventing the assembly of PD-1:PD-L1 complex formation support the formation of a defective ternary complex as the mechanism of action of CA-170.

Based on the above concepts, the interaction studies were carried using a two-point HSQC method using ^13^C, ^15^N Asn-labeled CA-170, and CHO-K1 cells overexpressing full-length human PD-L1 (Fig. 3a). The representative first FIDs of ^13^C-^1^H HSQC spectra recorded with delay and without the delay from CHO-K1 cells treated with 10 μM CA-170 with the values of *I*/*I*_0_ and *T*_2_ is presented along with NMR pulse program in Supplementary Fig. 1. At lower concentrations, as expected for a ligand binding to cells expressing the target protein, lower weighted average *T*_2_ values were obtained indicating that CA-170 was mostly bound. With an increase in CA-170 concentration, a higher weighted average *T*_2_ values were obtained indicating a greater percentage fraction of unbound CA-170. In similar experimental conditions performed with CA-170 and parental CHO-K1 cells, with no measurable PD-L1 expression, the *T*_2_ values were larger and changed marginally with no dose-dependency compared to lower *T*_2_ values that increased in a dose-dependent manner in PD-L1 overexpressing CHO-K1 cells indicating a lack of binding of the ligand to the parental CHO-K1 cells (Fig. 3b, c, Supplementary Table 8). Together these findings confirm the specific interaction of CA-170 to PD-L1 overexpressed in the cellular context.

Since CA-170 shows potent blocking of PD-1 activity in functional assays and binding to PD-L1, we sought to determine if this binding results in the prevention of the PD-1: PD-L1 complex. In a widely used TR-FRET assay, while a PD-1 antibody was able to prevent the interaction of PD-1 with PD-L1 in a dose-dependent manner, CA-170 did not lead to prevention of complex formation even when tested at a very high concentration of 100 μM despite the use of protein components expressed in mammalian cells (Fig. 3d). This data is consistent with the recently published data that indicates the inability of CA-170 to prevent PD-1:PD-L1 complex formation^25–27^.

Taken together with the blocking of PD-L1 signaling as observed in functional assays and binding to PD-L1 in the cellular context without preventing the assembly of PD-1:PD-L1 complex support the formation of a defective ternary complex as the mechanism of action of CA-170 (Fig. 3e).

### CA-170 shows in vivo immune pharmacodynamics and efficacy

**Fig. 4:**
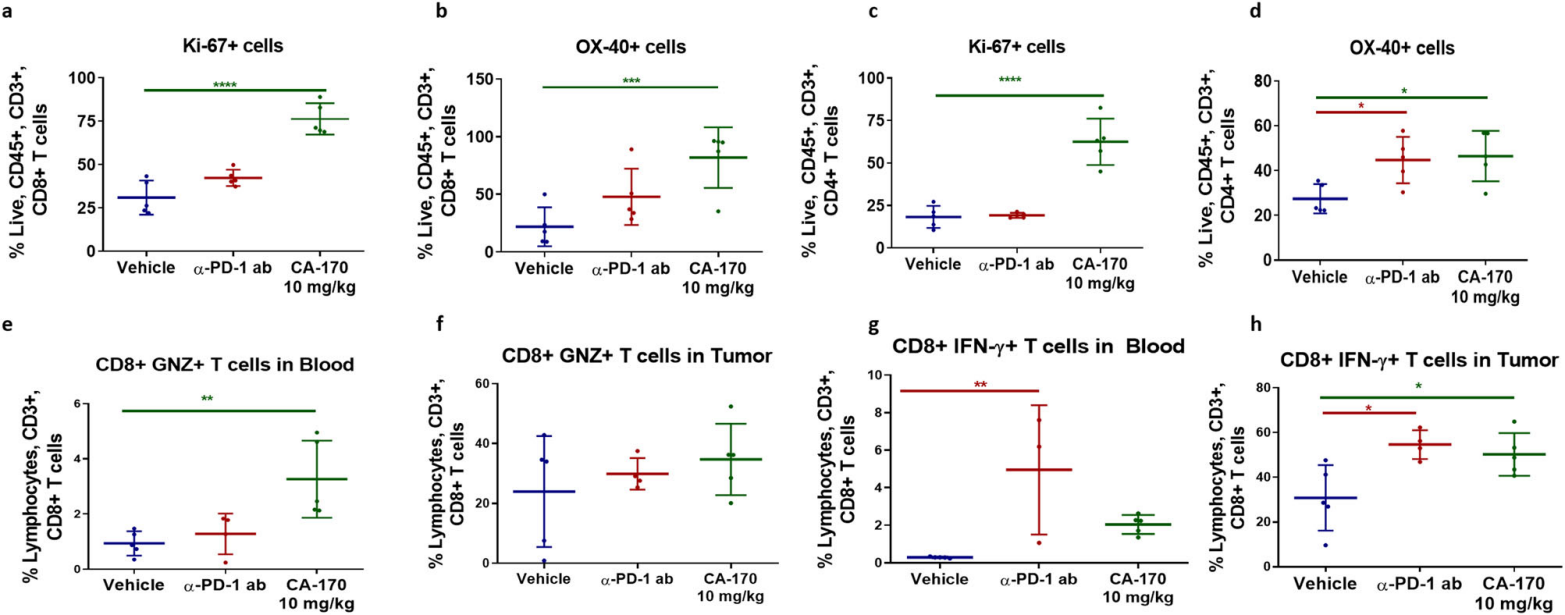
Immune PD of CA-170 in CT26 colon carcinoma model. CT26 tumor bearing mice were dosed with CA-170 at 10 mg/kg daily along with a vehicle control and a PD-1 antibody control (J43 Clone, 100 µg/animal) (*n* = 5). After 5 days of dosing the tumor infiltrating lymphocytes were labeled with Ki67 as a measure of proliferation and OX-40 as an indicator of T-cell activation. **a**–**b** Increase in the proliferating and activated CD8 + T cells. **c**–**d** Increase in the proliferating and activated CD4 + T cells. **e**–**f** Increase in granzyme B and **g**–**h** IFN-γ secreting CD8 + T cells in blood and tumor of MC38-tumor bearing mice dosed daily at 3 mg/kg for 7 days. *p < 0.05, **p < 0.01, ***p < 0.001, ****p < 0.0001 as calculated by one-way ANOVA (Dunnett’s multiple comparisons test).

Immune pharmacodynamic modulation upon dosing of CA-170 was analyzed in the immunogenic CT26 colon^31^ model. CA-170 treatment resulted in increased number of proliferating CD8 T cells (Fig. 4a) and CD4 T cells (Fig. 4c) as well as increased expression of co-stimulatory molecule OX-40 on CD4 and CD8 T cells (Fig. 4b, d) indicating immune-activation potential of CA-170. A significant increase in the intracellular levels of granzyme B in CD8 T cells in the blood was observed (Fig. 4e, f). Intracellular IFN-γ levels in CD8 T cells in the blood as well as tumors were found to be higher in CA-170 treated groups than that observed in the vehicle group (Fig. 4g, h).

**Fig. 5:**
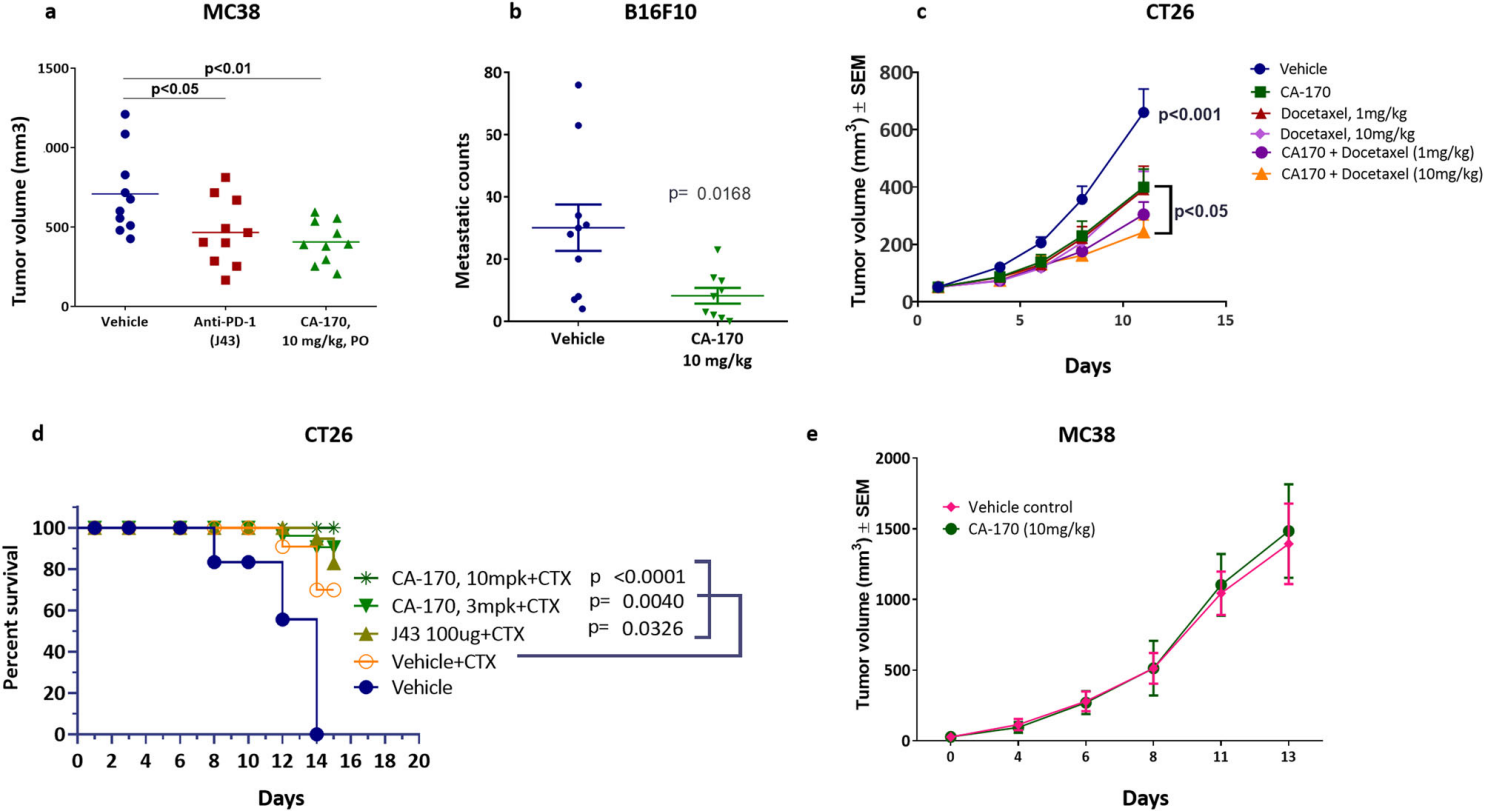
Anti-tumor efficacy of CA-170 in multiple mouse models. **a** Efficacy in MC38 model. C57B6 mice were injected with MC38 cells and received CA-170 at 10 mg/kg/day orally or PD-1 antibody (J43 clone) at 100 µg/animal/week subcutaneously for a period of 14 days starting from Day 5 (*n* = 10). On day 14 of dosing, statistically significant efficacy of 43% (*p* < 0.01) was observed with the group treated with CA-170 at 10 mg/kg, which was comparable to that of PD-1 antibody (tumor growth inhibition of 36%) (J43 clone) (*p* < 0.05). **b** Efficacy in B16F10 metastasis model. B16F10 cells were injected intravenously to C57B6 mice on day 0 and dosed with CA-170 (*n* = 9) or a vehicle control (*n* = 10) starting from day 1 orally at 10 mg/kg/day dose for a period of 14 days resulted in a 73% reduction in metastatic nodules (*p* < 0.01) as compared to a vehicle control. **c** Efficacy in CT26 model in combination with docetaxel. Balb/c mice were injected with CT26 cells and were treated with CA-170 at 10 mg/kg/day, docetaxel at 1 mg or 10 mg/kg/day or their combination for 12 days (*n* = 9). Group treated with CA-170 at 10 mg/kg showed 43% TGI while single agent docetaxel at 1 and 10 mg/kg doses exhibited 44% and 43% TGI, respectively. Combination group of CA-170 at 10 mg/kg and docetaxel at 10 mg/kg showed a statistically significant TGI of 68% (*p* < 0.05). **d** Efficacy in CT26 model. Balb/c mice were injected with CT26 cells and received CA-170 at 3 or 10 mg/kg/day orally or PD-1 antibody (J43 clone) at 100 µg/animal/week subcutaneously in combination with a single dose of cyclophosphamide (CTX) on the first day at 100 mg/kg (*n* = 9). Statistically significant survival advantage was observed when vehicle + CTX is compared with PD-1 antibody + CTX (*p* < 0.001), CA-170 (3 mg/kg) + CTX (*p* < 0.01) or CA-170 (10 mg/kg) + CTX (*p* < 0.001) groups. **e** Efficacy in an immunodeficient mouse model. Immunodeficient SCID beige mice were injected with MC-38 cells and animals received either a vehicle control or CA-170 (10 mg/kg/day) treatments for 14 days (*n* = 11) and there was no difference in the tumor volumes of the two groups. Student’s *t* test was used to determine the statistical significance in all in vivo experiments and the statistical significance is denoted as *p < 0.05, **p < 0.01, ***p < 0.001, ****p < 0.0001.

The efficacy of CA-170 was explored using three independent immunocompetent tumor models established in mice: the CT26 and MC38 colon carcinoma model and the B16F10 melanoma lung metastasis model. As a single agent CA-170 dosed orally once a day showed a significant tumor growth inhibition in MC38 (43%; Fig. 5a) and significant reduction in metastatic counts in B16F10 (73%; Fig. 5b) models, respectively. Many of the widely used therapeutic agents, such as docetaxel are reported to induce immunological cell death, which in turn leads to additive or synergistic anti-tumor activities in combination with checkpoint inhibitors^32^. In the CT26 colon carcinoma model, CA-170 showed an enhanced tumor growth inhibition in combination with docetaxel (68% TGI, *p* < 0.05; Fig. 5c) which is higher than TGI observed with docetaxel alone (43% TGI, *p* < 0.05; Fig. 5c).

Low dose of cyclophosphamide is known to reduce the Treg population and thereby enhance efficacy of immunomodulatory agents^33^. In the CT26 colon carcinoma model statistically significant survival advantage was observed when 3 mg/Kg or 10 mg/kg of CA-170 was combined with cyclophosphamide at 100 mg/kg (*p* < 0.001) indicating additive antitumor effect in CT26 colon carcinoma model (Fig. 5d). A significant increase in the tumor infiltrating CD8 T cells in the CA-170 in combination with cyclophosphamide group led to a favorable tumor suppressive CD8 T cell: FoxP3 ratio, thus accounting for the enhanced efficacy in this group (Supplementary Fig. 2).

To further confirm the requirement for immune-mediated mechanism for efficacy with CA-170, CA-170 was evaluated for its effect on the growth of MC38 tumors in severely immune-deficient (SCID beige) mice. There was no statistically significant difference between the tumor growth rate of the CA-170-treated group and the vehicle-treated group (Fig. 5e) in this immunodeficient model, confirming the dependency of CA-170 on a functional immune system for anti-tumor efficacy.

In all the in vivo pharmacological studies described above CA-170 dosing was well tolerated as evidenced by the lack of reduction in the body weights or clinical signs in any of the treatment groups during the study period.

## Discussion

Our efforts have resulted in an orally bioavailable small molecule checkpoint inhibitor CA-170. Unlike most widely followed approaches in which designed, or library of compounds are screened in a binding assay, we have utilized a functional assay in which the rescue of functionality inhibited by PD-L1. We reasoned that presentation of the target (PD-L1 in this case) would be in the native context such as appropriate post-translational modification, presentation of all the hot spots, as well as the interaction of the target of interest with accessory proteins, may also contribute to signaling. Such a notion is substantiated by a recent report highlighting discovery of a highly active PD-1 neutralizing antibody specifically targeting the glycosylation site^34^. Additionally, by monitoring the functional rescue of T cells from the effect of inhibitory ligands, it would be possible to identify compounds that would lead to inhibition of function without blocking the ligand–receptor interaction but perturbing the complex by binding to one of the partners in the complex. Specifically, the possibility of identifying a relatively polar compound targeting the solvent exposed grooves which are considered hard to target with small molecules^35^ can be identified. Because of the distinctiveness of our approach, we were also fully aware of the possibility that the compound identified by such a functional assay may behave differently than widely studied neutralizing antibodies or small molecules (typically identified and optimized using binding assays) in the in vitro assay systems.

Our functional assay utilizes the native cells and monitors proliferation and cytokine secretion from T cells (as expected in the therapeutic context), in which CA-170 shows the extent of maximum rescue (Emax) and potency similar to that observed with an anti-PD1 antibody. This is in contrast to published NFAT-reporter-based assay system, where antibodies show good potency and Emax, whereas small molecules showed substantially lower activity (biochemical to cellular potency difference of >100-fold in several instances and <1.5-fold induction of reporter activity) while CA-170 displayed no activity^25^, suggesting a sub-optimal nature of such an assay for characterizing small molecule inhibitors.

Unlike other reported small molecule PD-L1 antagonists^15,36^, CA-170 is relatively polar and thus likely interacts with the target of interest at a solvent exposed region. Published findings as well as our studies utilizing solution NMR have indicated a lack of binding of CA-170 to PD-L1^25^. The lack of definitive binding in these studies may also be due to the use of protein expressed in *E. coli* deficient in appropriate post-translational modifications and devoid of functional activity. Towards characterizing the interaction of CA-170 identified in a functional assay, we utilized cellular NMR spectroscopy using PD-L1 expressed in mammalian cells in the native context along with the parental cell line as a negative control. Cellular NMR data demonstrated dose-dependent increase in *T*_2_ values of CA-170 in the presence of PD-L1 overexpressed CHO-K1 cells compared to parental CHO-K1. The lower *T*_2_ values that increased upon increasing the CA-170 concentration indicate the binding of CA-170 to cellular PD-L1 under physiological environment. Although the glycosylation is important for the activity of PD-L1^34^, it is not clear which post-translation modification(s) (glycosylation and/or other) are important for the observed effect of CA-170 towards PD-L1. Unlike small molecules reported to induce dimerization of PD-L1 and disrupt complex formation, CA-170 did not inhibit PD-1:PD-L1 complex formation even when assays were done utilizing proteins expressed in mammalian cells^25–27^. This is in contrast to published data with other small molecule agents^27,37,38^ and antibodies^39,40^ which bind to the hot spots buried within the interface and not exposed to solvent to prevent interaction in a TR-FRET-based binding assay. CA-170 being substantially smaller than the full-length PD-1 protein (~90x smaller) and relatively polar, when bound to PD-L1 is expected to interact at a relatively small hydrophilic solvent exposed hotspot within the binding interface of PD1: PD-L1. Taken together with the effect of blocking the functionality, these observations are supportive of binding of CA-170 to critical residue(s) or hotspot on PD-L1 which by itself is not sufficient to prevent complex formation but leading to a functionally inactive ternary complex (Fig. 3e). In this context, it is important to note that a mechanism of action that does not involve the prevention of PD1-PDL1 complex formation has also been reported with functionally potent and pharmacologically active anti-PD-1 antibodies^12,13^.

Structural studies published earlier have indicated the presence of three hot spot pockets in PD-L1, binding to which can lead to inhibition of PD-1 signaling pathway^35^. Among these pockets, the hydrophobic amino acid residues are involved in hotspots 1 and 2, which are proposed to be ideal for binding to PD-L1 using conventional small molecules that exhibit considerable hydrophobicity^37^. The third hot spot is reported to be a solvent exposed with extended shallow groove with multiple hydrogen bond donors and acceptors and considered to be highly hydrophilic. Hence this is considered to be challenging to target via conventional small molecules.

CA-170 is derived from the linear peptide sequence SNT and the pharmacophore residues are attached non-contiguously in a non-peptidic heterocyclic 1,2,4-oxadiazole template with an insertion of urea bond between N and T. The sequence SNT is present contiguously in BC loop and non-contiguously within the sequence SNQT in CC′ loop and C′-strand of PD-1. Interestingly, importance of the BC loop of PD-1, specifically in the context of glycosylated protein has been highlighted in a recent publication^34^. Because of the optimal spatial disposition of amino acid side chains of S and T on a heterocyclic template with ample spacing of T in CA-170, CA-170 side chains likely occupy the space of SNQT from PD-1 and engage in the interaction with amino acids present in the third polar hot spot of PD-L1. This proposed interactions of CA-170 is expected to be similar to interaction of SNQT in CC′ loop and C′-strand of PD-1 in intimate contacts with residues of G-strand (A121, D122, Y123, and K124) and A strand (D26) in PD-L1 apart from the other interactions in PD-1:PD-L1 interface^41^. It is interesting to note that the residues D26 and D122 to R125 are also part of the proposed third hydrophilic hotspot of PD-L1, considered to be challenging to be targeted by the small molecules due to the lack of hydrophobicity^35^.

We had hypothesized that the minimum pharmacophores derived from the protein–protein interacting interface of PD-1 in our approach also has the potential to interact with other proteins belonging to the same immunoglobulin superfamily (such as VISTA, TIM3 etc.) with structurally similar grooves caused by pockets of sequence similarity^42^ resulting in the identification of either selective or spectrum-selective agents. Consistent with this hypothesis, CA-170 inhibits both PD-L1 and VISTA. This is a first report of a small molecule immune checkpoint inhibitor targeting two checkpoint proteins. PD-L1 and VISTA both belong to B7 family and have pockets of sequence and thus structural similarity^42^. It is likely that CA-170 derived from the shortened minimum pharmacophore derived from the interaction interface of PD-L1 and PD-1 recognizes the binding pockets or grooves conserved in VISTA. This dual active nature of CA-170 is analogous to small-molecule kinase inhibitors targeting more than one target vs exquisite selectivity of an antibody to a kinase target.

VISTA is an independent checkpoint protein that is reported to be ubiquitously present on immune cells within the tumor. VISTA is also known to be upregulated on tumor cells in cancers such as mesothelioma, ovarian cancer, gastric cancer, and NSCLC^43–46^, and upregulated in prostate cancer upon treatment with anti-CTLA4 antibody^8^. Preclinical studies using antibodies as well as knockout mice have demonstrated that the combination blockade of PD-L1 and VISTA leads to additive or synergistic antitumor activities when these two pathways are blocked simultaneously^47^. Therefore, simultaneous blockade of PD-L1 and VISTA could be beneficial for cancer immunotherapy, and a number of antibodies targeting VISTA are in pre-clinical development for potential use in combination with anti-PD-1/PD-L1 antibodies in the clinic. Because of its ability to block both PD-L1 and VISTA, CA-170 can also be potentially positioned in clinical indications where dual blockade is necessary to achieve the optimal therapeutic benefit.

CA-170 displayed good oral bioavailability and a relatively shorter PK exposure with a *t*_1/2_ of 3.4 h. A shorter PK drug exposure could be an advantage in managing immune-related toxicities. Antibodies targeting PD-1 and PD-L1 show immune-related toxicities albeit at lower frequency compared with anti-CTLA4 antibodies. Sustained target inhibition as a result of a long half-life (>15–20 days) and approximately 70% target occupancy for months are likely contributing to severe irAEs observed in the clinic with antibodies targeting immune checkpoint proteins. While the current management of irAEs with anti-PD-1/PD-L1 includes treatment cessation along with administration of steroids and or anti-TNF agents, the treatment cessation without the need for administering other agents, could be possible with CA-170.

In the completed Phase 1 study CA-170 was orally dosed from 50 to 1200 mg. CA-170 showed acceptable safety profile and exhibited generally dose proportional plasma exposure with a *T*_1/2_ of ∼4–9.5 h. Evidence of peripheral T-cell activation was observed with an increased proportion of circulating CD8 + and CD4 + T cells expressing activation markers, CD69, Granzyme B and OX-40 (CD134), following oral dosing^48^. In Phase 2 studies CA-170 has shown encouraging clinical activity which includes ORR of 30% in Classical Hodgkin lymphoma (based on Lugano criteria), and a CBR of >85% at a daily dose of 400 mg and PFS of 19.6 weeks (compared to PFS of ~8 weeks with best supportive care in a cross-study comparison) in advanced (stage 4) non-squamous NSCLC^49,50^.

In summary, we have developed an orally bioavailable small molecule dual inhibitor of PD-L1 and VISTA that exhibits desirable pharmacological and safety features. The distinct approach used here could potentially be applicable for identification of small molecule checkpoint inhibitors targeting other checkpoint proteins either singly or in combination. Compared to approved antibodies in the clinic, CA-170 offers the convenience of oral dosing, shorter half-life to better management of adverse events, dual inhibition of PD-L1 and VISTA to potentially achieve anti-tumor response in patients with the activation of both these checkpoint pathways. Ease of synthesis of CA-170 compared to antibodies that require recombinant mode of production may also lead to lower cost of goods. Based on the desirable overall profile, CA-170 has now progressed to the clinic (Phase 1 completed; ClinicalTrials.gov Identifier: NCT02812875, and Phase 2b/3 starting soon).

## Methods

### Antibodies and cell lines

Supplementary Table 1 in the supplementary information file lists the antibodies used in this study. Unless stated otherwise, all cell lines were obtained from ATCC and tested for mycoplasma using Venor GeM Mycoplasma Detection Kit (Sigma-Aldrich, MP0025). Cells were cultured at 37 °C in a CO_2_ incubator according to the provider instructions. Typically, cells were kept in culture for a minimum of two passages prior and a maximum of 20 passages when the experiments were performed.

### Animal studies

All animal experiments were approved by the Institutional Animal Ethical Committee based on the Committee for the Purpose of Control and Supervision on Experiments on Animals (India) guidelines.

### Blood collection from healthy volunteers

Blood collection for experimental purpose was initiated only after written approval from Suraksha Independent Ethics Committee (SIEC), India and written informed consent from healthy voluntary donors.

### Synthesis of peptides, peptidomimetics, and small molecules

Relevant synthetic information and references are covered for the test compounds in the supplementary information file under the section Supplementary methods.

### PD-L1/L2 functional assays using human PBMCs or mouse splenocytes

The ability of peptides, peptidomimetics, and small molecules were analyzed for their ability to rescue T-cell proliferation or IFN**-γ** release from the inhibitory activity of PD-L1 or PD-L2 in a splenocyte/PBMC-based functional assay as per the protocols reported in publication^20^. Human PBMCs isolated from individual donors in which proliferation and IFN**-γ** release were inhibited by PD-L1 (a minimum inhibition of 50% for proliferation and 70% for IFN**-γ** release) were used in the assays for analyzing the test agents. For all in vitro assays, test agent stock was prepared in water and diluted further in the assay media to achieve test concentrations. Each experimental condition was carried out in triplicates. Percent rescue for a given test compound concentration was calculated by normalizing individual percent test agent proliferation or IFN**-γ** release values to percent anti-CD3 and anti-CD28 antibodies stimulated proliferation or IFN**-γ** release. EC_50_ values were derived by plotting transformed data into non-linear fit in sigmoidal dose–response curve using GraphPad Prism software (Version 7).

### Plasma and metabolic stability of test compounds

Test compounds were tested for plasma stability (test concentration of 10 μM) as per the protocols reported in publication^20^.

### Pharmacokinetics in Balb/c mice

Twelve male CD-1 mice (9–11 weeks old) were used in each dosing groups [Group 1, 3 mg kg^−1^ per intravenous (IV) administration; Group 2, 30 mg kg^−1^ per oral (PO) administration]. Animals in Group 1 were administered with 5% w/v dextrose in water and Group 2 were administered with corresponding formulation listed in the Supplementary Table 6 through oral routes. Blood samples (approximately 40 μl) were collected under light isoflurane anesthesia from submandibular at predose, 0.08 (IV), 0.25, 0.5, 1, 2, 4, 8, and 24 h using sparse sampling technique. Plasma samples were separated by centrifugation of whole blood and stored below −70 °C until bioanalysis. All samples were processed for analysis by protein precipitation and analyzed with fit-for-purpose LC–MS/MS method. From intravenous and PO administration, pharmacokinetic parameters such as plasma concentration after injection (*C*_0_ min), the area under the concentration−time curve (AUC), volume of distribution (*V*_d_) and clearance (CL), maximum plasma concentration (*C*_max_), time to reach maximum plasma concentration (*T*_max_), and % fraction absorbed were obtained using WinNonlin 8. 3.

### CTLA4-B7.1 functional assay

CA-170 was evaluated for its potential impact on B7.1 mediated immune-inhibitory interaction with CTLA4 by monitoring the rescue of release of IL-2 in the presence of recombinant CTLA4-Ig and B7.1 in T cells stimulated with phytohemagglutinin (PHA). Briefly, Jurkat T cells (0.25 × 10^5^ cells/well of a 96-well plate) grown and starved in serum-free RPMI1640 media for 2 h were stimulated with PHA (10 µg/mL) and B7.1 (500 ng/mL) in the presence of recombinant human CTLA4-Ig (200 ng/mL) and different concentrations of CA-170 and cultured for 48 h at 37 ^o^C. After 48 h of cell culture, supernatant was analyzed for IL-2 by ELISA. Anti-CTLA4 antibody and isotypes were used as controls.

### CD28-B7.1 functional assay

Impact of CA-170 on immunostimulatory interaction was examined in a Jurkat T-cell-based assay in which the ability of B7-1 to induce production of IL-2 was monitored upon stimulation with PHA. Briefly, Jurkat T cells (0.25 × 10^5^ cells/well of a 96-well plate) grown and starved in serum-free RPMI1640 media for 2 h were stimulated with PHA (5 µg/mL) and B7.1 (1.0 µg/mL) and different concentrations of CA-170 and cultured for 48 h at 37 °C. After indicated time of incubation, culture supernatant was analyzed for IL-2 by ELISA. Anti-CD28 antibody and isotypes were used as controls.

### VISTA functional assay

The ability of CA-170 to rescue VISTA-mediated inhibition of IFN-γ release from anti-CD3/CD28-stimulated human PBMC was used to monitor the effect of CA-170 on VISTA functionality. Briefly, 96-well cell culture plates were pre-coated with recombinant human VISTA (2.5 µg/mL) and anti- human CD3 (2.5 µg/mL) overnight at 4 °C. Anti-human VISTA and isotype control antibodies were either coated along with the VISTA or incubated for 30 min next day before addition of cells. On the next day, plates were incubated with CA-170 for 30 min at different concentrations. Isolated PBMC (0.1 × 10^6^ cells/well) and anti-human CD28 antibodies (1 µg/mL) were added to the wells. The culture was further incubated for 72 h at 37 °C with 5% CO_2_. After 72 h of incubation the cell culture supernatants were collected after brief centrifugation at 200 *g* x 5 min at 4 °C and processed for human IFN-γ measurement by ELISA following manufacturer’s protocol (R&D Systems; DY-285). Percent rescue of IFN-γ release was calculated by normalizing individual percent test agent IFN**-γ** release values to percent anti-CD3 and anti-CD28 antibodies stimulated IFN**-γ** release. EC_50_ values were derived by plotting transformed data into non-linear fit in sigmoidal dose–response curve using GraphPad Prism software (Version 7).

### Cytokine induction in human PBMCs

To determine the potential for any unforeseen T-cell agonist activity in humans, a study was conducted where human PBMCs from healthy donors were pre-cultured either at normal density (2 × 10^5^ cells/well) or at high density (1 × 10^7^ cells/mL) in vitro to increase cell–cell contacts and immune scanning as described^24^ and then treated with CA-170 (10 nM to 1000 nM) IL-2, TNF-α and IFN-γ cytokine release was measured in the culture supernatants at 24 and 48 h. Human PBMCs stimulated with anti-CD3 alone or anti-CD3 + anti-CD28 antibodies served as positive controls for cytokine release in this assay.

### CEREP 80 panel assay

CEREP 80 panel assay was carried out by Eurofins Cerep Panlabs facility at France. The data is tabulated in Supplementary Table 4. A series of binding assays was carried out to understand the off-target toxicity of CA-170. CA-170 was tested at 10 μM test concentration in duplicates. Compound binding was calculated as a % inhibition of the binding of a radioactively labeled ligand specific for each target. In each experiment the respective reference compound was tested concurrently with CA-170, and the data were compared with historical values determined at Eurofins. The experiment was accepted in accordance with Eurofins validation standard operating procedure. The results are expressed as a percent of control specific binding; (measured specific binding / control specific binding) × 100 and as a percent inhibition of control specific binding; 100−(measured specific binding / control specific binding) × 100 obtained in the presence of CA-170. The IC_50_ values (concentration causing a half-maximal inhibition of control specific binding) and Hill coefficients (nH) were determined by non-linear regression analysis of the competition curves generated with mean replicate values using Hill equation curve fitting.

### Generation of PD-L1 overexpressing CHO-K1 cells

HEK293T cells were transfected with plasmid (pLVX puro-PD-L1; full-length human PD-L1) using Lenti-X packaging single shots (Takara Bio, Cat. Nos. 631275) as per the recommended kit protocol in order to generate lentiviral particles. The supernatant collected after 48 h containing the lentiviral particles was used for lentiviral transduction of CHO-K1 cells. The expression of PD-L1 on the CHO-K1 cells was determined by flow cytometry. Limiting dilution cloning was performed to isolate single clones of CHO-K1 cells expressing high levels of PD-L1.

### NMR studies to characterize the binding of CA-170 to PD-L1

PD-L1 transfected CHO-K1 cells were cultured in Ham’s F12 K medium (Cat#21127-022, Gibco) containing penicillin & streptomycin antibiotic solution and 10% FBS (cat#SH30071-03, HyClone) along with 4 µg/mL of puromycin (Cat#A11138-03, Gibco), and were grown at 37 °C, 5% CO_2_ in a humidified atmosphere in tissue culture treated flask. 80–90% confluent cells were detached with PBS containing 2 mM EDTA, counted and washed twice with phosphate-buffered saline, pH 7.4 and re-suspended in PBS(cat#14190-144 Gibco) at a density of 8 million cells per mL. The cell suspension in PBS was transferred in a 5 mm Norell NMR tube containing 5% D_2_O and NMR experiments were performed under ligand titration. Cells remained viable under these experimental conditions as assessed by Trypan Blue staining method.

NMR experiments were carried out using Bruker 800 MHz NMR spectrometer fitted with 5 mm TCI cryoprobe. The experiment was performed over 4 × 10^6^ cells in 95% 1 X PBS, pH 7.4, after adding 5% (v/v) D_2_O at constant temperature 298 K employing the pulse sequence shown in Supplementary Fig. 1a. The FID obtained was processed employing TOPSPIN software version 3.5. The interaction studies of CA-170 and overexpressed cells were carried out using a two-point HSQC method by recording the first FID. Briefly, during the transfer of polarization from ^1^H to ^13^C in the forward INEPT part of the 2D [^13^C, ^1^H] HSQC experiment, an additional delay of 16 ms is introduced on the proton channel during which the signal decays due to transverse relaxation. A second experiment is recorded with this delay set to 4 µs, the experimental parameters used to record these two spectra are given in Supplementary Table 7. A representative NMR spectrum of superimposed first FID of modified 2D [^13^C, ^1^H] HSQC of CA-170 treatment with PD-L1 overexpressed CHO-K1 cells is shown in Supplementary Fig. 1b.

The signal at any point of time is related to the initial intensity as follows.

$$I = I_{{\rm{o}}}{\rm{exp}}\,(-\varDelta {t}/{T}_{2})$$

Where Δt is the difference between initial and final time points and I and Io are the final and initial intensity respectively. The transverse relaxation time (T_2_) of the ligand protons is calculated from the signal intensity ratio obtained from the above experiments by using the following equation which can be obtained from the above relation (Supplementary Table 8).

$$T_{2}={\rm{Relaxation}}\,{\rm{delay}}\,( \tau ) / - {\rm{log}} \, ({I/I}_{0})$$

where *I* = Area of the peak with delay (16 ms delay); *I*_0_ = Area of the peak without delay (16 μs delay).

### TR-FRET assay

The assay was performed using LANCE Ultra TR-FRET binding assay. To determine the half maximal inhibitory concentration (IC_50_) of tested compounds, they were solubilized in water and measurements were performed on individual dilution series. His-tagged human recombinant PD-L1 protein (Sino Biologicals, Cat#10084-H08H) at 20 nM final concentration was incubated at 25 °C temperature with indicated concentration of test compound for 15 min in a white Lumitec high-binding, 384-well plate (Grenier, Cat #781074). Human recombinant PD-1 Fc chimera protein from R&D system (Cat # 1086-PD) was then added at 5 nM final concentration and the mixture was further incubated for 15 mins. LANCE Eu-anti-human IgG from Perkin Elmer, (Cat# AD0212) at 0.5 nM final concentration and Surelight Allophycocyanin-6-His from Perkin Elmer (Cat# AD0059H) at 20 nM final concentration was added to the wells. Plates were incubated for 1 h prior to reading in Perkin Elmer WALLAC 1420 Multilabel Counter Victor 5 at the wavelength Ex: 340 nm Em: 615 nm and 665 nm. Collected data was background subtracted on the negative control, normalized, and analyzed using non-linear regression analysis to determine the IC_50_ value by GraphPad Prism Version 8.

### Analysis of the immune pharmacodynamic effect in a syngeneic tumor model

For the measurement of immune pharmacodynamics, CT26 cells were subcutaneously implanted into 5 to 7 weeks old male Balb/c mice by injecting 1 million CT26 tumor cells/ animal. When tumor volume reached ~200mm^3^, animals were randomized and allocated to three treatment groups (*n* = 5). Similarly, for the measurement of immune pharmacodynamics in MC38 model, MC38 cell lines were subcutaneously implanted into 5 to 7 weeks old male C57B6 mice by injecting 0.5 million cells/animal. Dosing was initiated when tumor volume reached ~100 mm^3^. In both tumor models, mice were orally treated daily with vehicle and CA-170 (10 mg/kg) for 5 days. As an additional control, anti-PD1 antibody (100 μg/animal, clone J43) was dosed intraperitoneally on day 1. After 24 h of last dosing, animals were sacrificed, and blood samples and tumor tissues were collected from each animal and processed for PD evaluation using antibodies shown in Supplementary Table 1 and specific methods reported in publication^51^ and analyzed by FACS. The gating strategy is detailed in Supplementary Fig. 3.

### Antitumor efficacy in syngeneic models

For MC38 syngeneic colon carcinoma subcutaneous model, 0.5 × 10^6^ MC38 cells (Source: NCI/NIH, kind gift from Dr. Michael Pollack) were injected subcutaneously to the left flank of the C57B6 mouse (In-house) or SCID Beige mice (Source: Harlan, France) on day 1 and dosing was initiated when average tumor volume reached ~100 mm^3^. Animals were randomized based on tumor volumes and treated with either a vehicle control or CA-170 at indicated doses for 14 days. Anti-PD1 antibody (Clone J43 from BioXcel) was dosed intraperitoneally at 100 µg/animal once every week. Treatment efficacy was assessed in terms of the effects of the test substances on the tumor volumes of treated mice relative to vehicle-treated mice. Tumor growth curves of test and control groups using the mean tumor volumes were drawn, percent tumor growth inhibition (%TGI) defined as the ratio of the average tumor volumes of treated versus control group multiplied by 100.

Studies in syngeneic models including B16 melanoma metastasis model and CT26 colon carcinoma subcutaneous model were performed as described^1^. For the Docetaxel combination experiment in CT26 model, indicated dosage of docetaxel (Source: Docetere® injection by Dr Reddy’s Laboratories) was dosed intraperitoneally to animals. For the CA-170 combination groups, CA-170 (10 mg/kg/day, oral) was dosed ~10 min after docetaxel. Animal grouping, treatment efficacy assessment and %TGI calculations were performed as mentioned above. For the CT26 experiment with cyclophosphamide, 100 mg/kg of cyclophosphamide was dosed once on day 5 intraperitoneally after cell implantation (first day of dosing for all other groups). Anti-PD1 antibody (Clone J43 from BioXcel) was dosed intraperitoneally at 100 µg/animal once every week.

### Statistics and reproducibility

Information regarding center values, error bars, number of replicates or samples, number of independent experiments, and statistical analyses are described in the corresponding figure and table legends. Experiments were not blinded, and sample sizes were not predetermined using statistical analyses.

### Reporting summary

Further information on research design is available in the Nature Research Reporting Summary linked to this article.

## Supplementary information

Supplementary Information

Supplementary Data 1

Description of Supplementary Files

Reporting Summary

## Data Availability

The source data underlying Figs. 1, 2, 3, 4, 5, and 6 are provided in Supplementary Data 1. The data supporting the findings of this study are also available from the corresponding author upon reasonable request.
