# Supplementary material for: PD-1 derived CA-170 is an oral immune checkpoint inhibitor that exhibits preclinical anti-tumor efficacy
Source: Commun Biol. 2021 Jun 8;4:699. doi: 10.1038/s42003-021-02191-1 (PMC8187357; doi:10.1038/s42003-021-02191-1)
Supplement: Supplementary file 1 — Supplementary Information [file 42003_2021_2191_MOESM1_ESM.pdf]

PD-1 derived CA-170 is an oral immune checkpoint inhibitor that exhibits preclinical anti-tumor efficacy

Pottayil G. Sasikumar[1], Naremaddepalli S. Sudarshan[1], Srinivas Adurthi[1], Raghuveer K Ramachandra[1], Dodderi S. Samiulla[1], Anirudha Lakshminarasimhan[1], Anuradha Ramanathan[1], Talapaneni Chandrasekhar[1], Amit A. Dhudashiya[1], Sumalatha R. Talapati[1], Nagesh Gowda[1], Sreenivasulareddy Palakolanu[1], Jiju Mani[1], Bandi Srinivasrao[1], David Joseph[2], Nigam Kumar[1$], Rashmi Nair[1], Hanudatta S Atreya[2$], Nagaraj Gowda[1] and Murali Ramachandra[1]*

[1]Aurigene Discovery Technologies Limited, Bangalore 560100, Karnataka, India

[2]NMR Research Centre, Indian Institute of Science, Bangalore 560012, Karnataka, India

$-Deceased

* Corresponding Author

SUPPLEMENTARY INFORMATION

SUPPLEMENTARY FIGURES

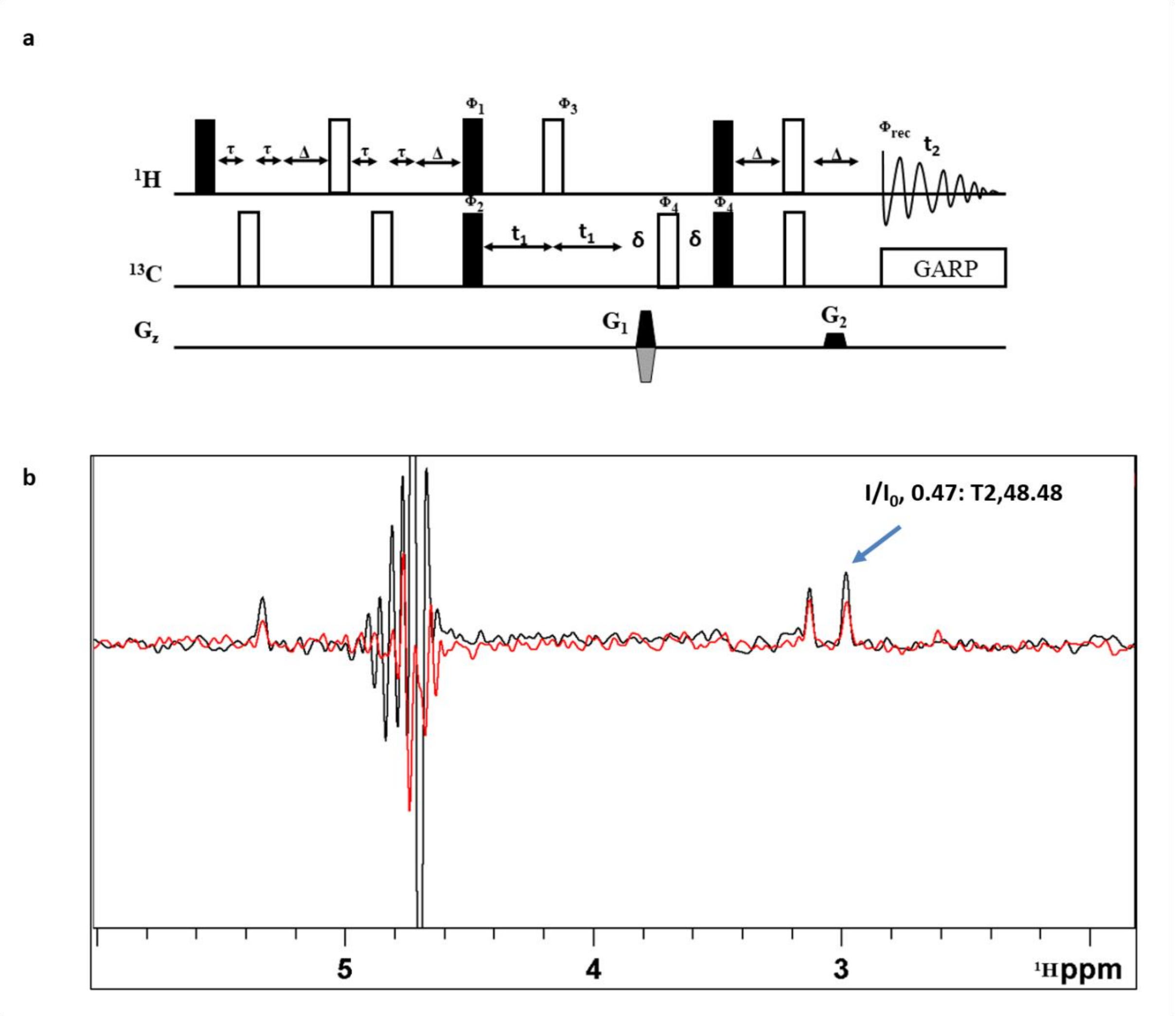

Supplementary Figure 1: P**ulse program and representative NMR spectrum**. **a.** Pulse scheme of the modified [^{1}H,^{13}C] HSQC for measuring T_2 relaxation values. The filled rectangles represent hard 90° pulses, the open rectangles represent hard 180° pulses and the filled trapezoids represent the gradient pulses. The delays Δ = 1/4JCH and τ is set as 4 us for experiment without delay and as 4 ms for experiment with delay. Phase cycling: Φ1 = y; Φ2 = x, -x; Φ3 = x, x, -x, -x; Φ4 = x, x, x, x, -x, -x, -x, -x Φrec (receiver)= x, -x, x, -x, -x, x, -x, x and the gradient strengths were with ratio of G1:G2 as 3.98:1. **b**. Representative NMR spectra of superimposed the first FID of modified 2D [^{13}C, ^{1}H] HSQC spectra of CA-170 treatment with PD-L1 overexpressed CHO-K1cells; black (without delay), Red (with delay) at 10 µM concentration. For the peak shown with an arrow, the calculated I/I0 and their corresponding T_2 values are shown.

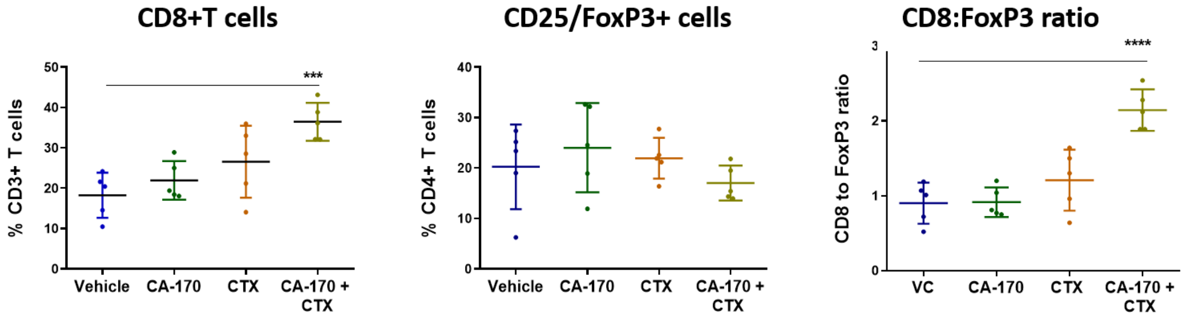

Supplementary Figure 2: **Effect of CA-170 alone and in combination with CTX on CD8/FoxP3 ratio**. No significant change in the levels of tumor-infiltrating Tregs were observed after administration of CA-170 in CT26 tumor model, as observed by the measuring CD4+CD25+FoxP3+ cells. However, an increase in the number of infiltrating CD8+ T cells increased in CA-170 + CTX treated group, led to a favourable increase in the ratio of CD8+T cells to Tregs increased significantly.

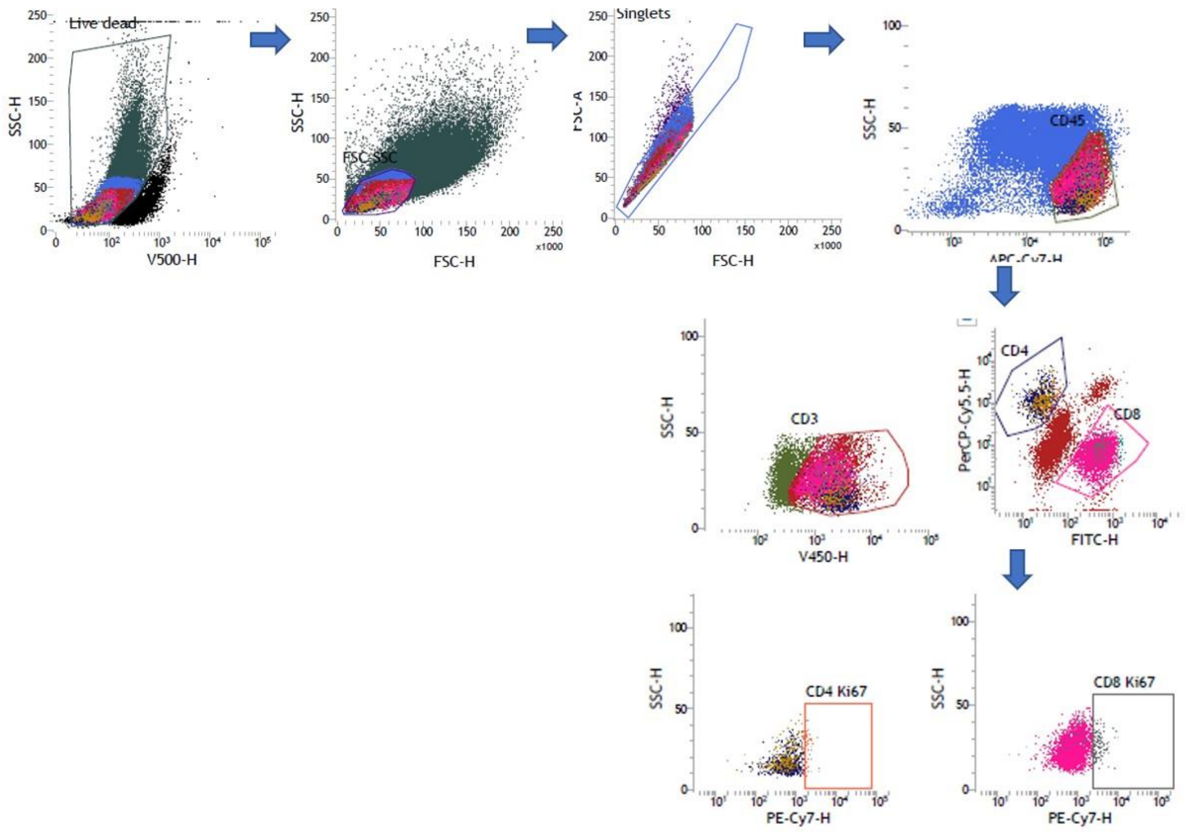

Supplementary Figure 3: **Gating Strategy used to analyze flow cytometry data:** Cells isolated from the tumor were acquired on BD FACS Verse flow cytometer. Cells were distinguished by means of a live-dead stain, followed by gating singlets, CD45 + cells, CD3+ cells, further into CD4+ and CD8+ cells. Ki67 and OX-40 was analyzed in each of these individual T cell populations.

SUPPLEMENTARY TABLES

Supplementary Table 1: List of Antibodies and major reagents used in the present study

Type	Reagent	Source and catalog number
Antibody	Anti-PD1, Clone J116	eBioscience, 16-9989-38
	Anti-mouse PD1, Clone J43	eBioscience, 16-9985-85
	Anti-VISTA	R&D systems, MAB7126
	Anti-CTLA4 Clone14D3	eBioscience, 16-1529-82
	Anti-CD28	eBiosciences, 16-0289
	Anti-CD3	eBiosciences, 16-0039
	Anti-mouse CD3	eBiosciences, 16-0032
	Anti-mouse CD28 antibody	eBiosciences, 16-0281
	Anti-CD4	eBiosciences, 45-0042-82
	Anti-CD8	eBiosciences, 17-0081, 11-0081-82
	Anti-CD45	Biolegend, 103116
	Anti-Ki67	Biolegend, 652426
	Anti-OX-40	Biolegend, 119411
	Anti-FoxP3	eBiosciences, 17-5773-82
	Anti-human PD-L1 –PeCy7 antibody	e-Bioscience, 25-5983-42
Recombinant proteins	Anti-mouse PD1, Clone J43	eBioscience,16-9985-85
	Human PD-L1	R&D systems, 156-B7-100
	Human PD-L2	R&D systems, 1224-PL-100
	Human VISTA	R&D systems, 126-B7
	Mouse -PD-L1	R&D Systems, 1019-B7-100
	Mouse -PD-L2	R&D Systems, 1022-PL-100
	Monkey – PD-L1	Sinobiological, 90251-C02H
	Monkey – PD-L2	Sinobiological, 90249-C02H
	Human CTLA4-Fc	R & D systems, 325-CT
	Human B7.1	R &D Systems, 140- B1
Other	PHA	Sigma, L1668
	CFSE	eBioscience 65-0850-85
	Human IFN-γ Duo set ELISA kit	R&D Systems, DY-285
	Mouse IFN-γ Duo set ELISA kit	R&D Systems, DY-485
	Lenti-X™ Packaging Single Shots	Takara, 631275

Supplementary Table 2: In vitro rescue of proliferation and IFN-γ production by T cells in human PBMCs stimulated in the presence of the exogenous immune suppressers PD-L1, PD-L2, and VISTA. Data are presented as mean ± S.D. of n = 3 or 4 biologically independent samples. N/C= not calculable, N/T= not tested

Test Compound	Human PBMC Proliferation Rescue EC_{50} (nM)		Human PBMC IFN-γ Rescue EC_{50} (nM)		
	PD-L1 (n=3)	PD-L2 (n=3)	PD-L1 (n=3)	PD-L2 (n=3)	VISTA (n=4)
CA-170	35 ± 6	51.5± 31.6	66.1± 23.2	80.9± 27.2	82.9± 37.1
Anti-PD-1 antibody (clone J116)	29.1± 8.4	22.8± 5.5	29.9± 17.1	35.8± 15.2	N/T
Anti-PD-1 isotype ctrl.	N/C	N/C	N/C	N/C	N/T
Anti-VISTA antibody (clone 730802)	N/T	N/T	N/T	N/T	53.9± 42.8
Anti-VISTA isotype ctrl.	N/T	N/T	N/T	N/T	N/A

Supplementary Table 3: In vitro rescue of proliferation and IFN-γ production by T cells in mouse splenocytes and monkey PBMCs stimulated in the presence of the exogenous immune suppressors PD-L1 or PD-L2. Data are presented as mean ± S.D. of n = 2 biologically independent samples for mouse splenocytes and n=1 for monkey PBMCs.

Test Compound	Mouse Splenocyte				Monkey PBMC			
	Proliferation Rescue EC_{50} (nM) (n=2)		IFN-γ Rescue EC_{50} (nM) (n=2)		Proliferation Rescue EC_{50} (nM)		IFN-γ Rescue EC_{50} (nM)	
	PD-L1	PD-L2	PD-L1	PD-L2	PD-L1	PD-L2	PD-L1	PD-L2
CA-170	15.4 ± 1.3	40.2 ± 10.5	34.65 ± 1.1	78.35 ± 13.9	34.05	36.78	97.77	154.6
Anti-PD-1 antibody (clone J43)	22 ± 6.9	19.45 ± 10.8	20.55 ± 11.2	101.5 ± 50.7	90.03	84.56	28.83	67.97

Supplementary Table 4: **CA-170 did not induce cytokines in an *in-vitro* cytokine storm assay**. Human PBMCs from healthy donors were pre-cultured at high density ($1x10^7$ cells/ml) for 48 hr to increase cell-cell contacts and immune scanning. The cells were then harvested and treated with CA-170 (10nM to 1000nM). IL-2, TNF-α and IFN-γ cytokine release was measured in the culture supernatants at 24 and 48 hours. Human PBMCs stimulated with anti-CD3 or anti-CD3+anti-CD28 antibodies served as positive controls for cytokine release in this assay. Two donors were used in this study one male and one female; there was no gender difference observed in the cytokine response in this study. Data tabulated is the representative data from a male donor (BLoD = Below Limit of Detection). Cytokine levels determined in PBMCs from the same donor cultured at a normal density of ($1 x 10^5$ cells/well) for 24 and 48 hr for comparison.

	**High density**			**Normal density**		
**Treatment Group**	**IL-2 (pg/ml) +/-SD**	**IFN-γ (pg/ml) +/-SD**	**TNF-α (pg/ml) +/-SD**	**IL-2 (pg/ml) +/-SD**	**IFN-γ (pg/ml) +/-SD**	**TNF-α (pg/ml) +/-SD**
	**24 hours**	**24 hours**	**24 hours**	**24 hours**	**24 hours**	**24 hours**
None	BLoD	9.9+/-1.7	95 +/-2.0	3.2+/-1.2	BLoD	44 +/-0.4
Anti-CD3	223 +/-3.0	137 +/-7.3	764 +/-20	346 +/-21	305 +/-4.6	392.9 +/-9.1
Anti-CD3/CD28	588+/-27	204 +/-11	1002 +/-29	861+/-47.7	455 +/-26.8	530.2 +/-51.8
CA-170 (10nM)	6.6 +/-2.6	2.8 +/-1.9	80.5 +/-9.1	3.9 +/-1.4	BLoD	47.1 +/-1.7
CA-170 (50nM)	3.6 +/-0.7	1.5 +/-1.0	70.9 +/-8.4	3.5 +/-0.3	BLoD	42.8 +/-3.4
CA-170 (100nM)	6.2 +/-1.7	1.5 +/-0	103.5 +/-8.5	4.2 +/-0.8	BLoD	39.8 +/-2.5
CA-170 (250nM)	1.5 +/-1.1	4.0 +/-0.7	93.1 +/-4.5	5.6 +/-1.1	BLoD	37.8 +/-2.5
CA-170 (500nM)	5.3 +/-2.5	6.8 +/-1.5	79.2 +/-1.8	4.8 +/-1.6	BLoD	41.5 +/-4.7
CA-170 (1000nM)	11.3 +/-0.6	5.8 +/-1.4	101.6 +/-9.9	5.8 +/-1.3	BLoD	47.9 +/-3.3

Supplementary Table 5: CEREP 80 panel assay. CA-170 binding at 10 µM was calculated as a % inhibition (or stimulation for assays run in basal conditions) of the binding of a radioactively labeled ligand specific for each target. Results showing an inhibition higher than 50% are considered to represent significant effects of the test compounds. Results showing an inhibition (or stimulation) between 25% and 50% are indicative of weak to moderate effects. Results showing an inhibition (or stimulation) lower than 25% are not considered significant and mostly attributable to variability of the signal around the control level. Low to moderate negative values are considered to have no real meaning and are attributable to variability of the signal around the control level.

Target		% Inhibition of Control Specific Binding
Adenosine	A_{1}, A_{2A}, A_{3}:	≤26
Adrenergic	α_{1}, α_{2}, β_{1}, β_{2}	≤6
Benzodiazepine	BZD (peripheral), BZD (central)	≤5
Glutamate	PCP	5
Cannabinoid	CB1	12
Dopamine	D1, D2S, D3, D44, D5	≤14
Cholecystokinin	CCK1, CCK2	<0
Galanin	GAL1, GAL2	≤12
GABA		11
Cl-Channel		2
Histamine	H1,H2	<0
Melatonin	MT1	5
Muscarinic	M1, M2, M3, M4, M5	≤12
Prostanoid	EP4, IP, TP	≤5
Cytokines	TNFα	<0
Growth factors	PDGF	6
purinergic	P2X, P2Y	≤3
Serotonin	5HT1A, 5HT1B, 5HT2A, 5HT2B, 5HT2C, 5HT3, 5HT5A, 5HT6, 5HT7	≤20
Sigma		20
Angiotensin -II	AT1, AT2	≤5
Bombesin	BB	<0
Bradykinin	B2	<0
CGRP		1
Chemokines	CCR1, CXCR2	1
Endothelin	ETA, ETB	≤9
Melanocortin	MC4	0
Neurokinin	NK1, NK2, NK3 (antagonist)	≤6
NeuropeptideY	Y1,Y2*	≤78
Neurotensin	NTS1	7

Opioid & Opioid like	DOP, KOP, MOP, NOP	≤9
Somatostatin	sst	1
VIP	PACAP,VIP1	≤9
Vasopressin	V1a	1
Nuclear receptors	GR, PPARγ	≤7
Ion Channels	Ca2+, Kv, Skca, Na2+	1
Amine transporters	DA, NE, 5HT	≤1

*50% is the most common cut-off value for further investigation, hence Y2 assay is taken for determination of IC_{50}. EC_{50} value not calculable. Concentration-response curve shows less than 25% effect at the highest validated testing concentration.

Supplementary Table 6: Oral formulation details of CA-170 and its precursors

Compound ID	Oral formulation composition
PM-13	0.5 % w/v Chitosan + 0.1 M Citric Acid + 1 % v/v NMP + 1 % v/v Tween 80 + 0.1 % w/v EDTA
PM-105	0.5 % w/v Chitosan + 0.1 M Citric Acid + 1 % v/v NMP + 1 % v/v Tween-80 + 0.1 % w/v EDTA
PM-209 and PM823	1 % v/v Tween 80 + 10 % v/v Capmul PG-8 + 5 % w/v HPßCD + 0.1 M citric acid + 0.1 % w/v EDTA.
PM-219	0.5 % v/v NMP + 5 % w/v HPßCD + 1 % w/v EDTA
CA-170	5 % v/v Solutol + 0.1 M citric acid + 0.1% w/v EDTA.

Supplementary Table 7: NMR acquisition parameters used for recording the first FID of the modified 2D HSQC experiment.

^{1}H Dimension		^{13}C Dimension	No. of Scans	Total time (min)
**Spectral Width (Hz)**	**Acquisition time (ms)**	**Spectral Width (Hz)**		
10416.667	196.6080	33330.836	512	19min 24sec

Supplementary Table 8: I/ I_0 ratio of each concentration ligand CA-170 and their corresponding T_2 values on treatment with PD-L1 over expressed cells and blank cells and their corresponding T_2 values.

**Trial 1**	**CA-170 Conc (µM)**	**I/I_0 (ppm)**	**T_2 (mS)**
**PD-L1 Overexpressed CHO-K1**	5.0	0.34	33.97
	7.5	0.40	41.00
	10.0	0.39	38.70
	12.5	0.53	57.00
	15.0	0.49	52.00
	20.0	0.57	66.12
	40.0	0.58	67.51
**Parental CHO-K1**	5.0	0.59	70.18
	10.0	0.59	69.86
	20.0	0.57	65.57
	40.0	0.60	72.39
**Trial 2**			
**PD-L1 Overexpressed CHO-K1**	5.0	0.34	29.67
	7.5	0.43	43.70
	10.0	0.45	46.10
	12.5	0.54	59.80
	15.0	0.55	61.50
	20.0	0.53	58.18
	40.0	0.55	61.50
**Parental CHO-K1**	5.0	0.56	63.50
	40.0	0.54	60.00
**Trial 3**			
**PD-L1 Overexpressed CHO-K1**	5.0	0.35	35.08
	7.5	0.49	51.60
	10.0	0.47	48.48
	12.5	0.46	47.05
	15.0	0.48	50.00
	20.0	0.51	55.36
	40.0	0.57	66.67
**Parental CHO-K1**	7.5	0.59	70.48
	12.5	0.57	64.00
	20.0	0.52	57.14
	40.0	0.57	66.66

Supplementary Methods

General Methods

Unless otherwise noted, reagents and solvents were obtained from commercial suppliers and were used without further purification. ^{1}H NMR spectra and ^{13}C-NMR were recorded on a Varian MR-400 NMR spectrometer operating at 400 MHz and 100 MHz respectively, and chemical shifts are reported in parts per million (ppm, δ) downfield from tetramethylsilane (TMS). Coupling constants (J) are reported in Hz. Spin multiplicities are described as s (singlet), br (broad singlet), d (doublet), t (triplet), q (quartet), and m (multiplet). Analytical High Performance Liquid Chromatography (HPLC) was performed on ZIC HILIC 200 A° column (4.6 mm × 250 mm, 5 µm), Flow rate: 1.0 mL / min. The elution conditions used are: Buffer A: 5 mmol ammonium acetate, Buffer B: Acetonitrile, Equilibration of the column with 90 % buffer B and elution by a gradient of 90 % to 40 % buffer B during 30 min. Preparative HPLC method: Preparative HPLC was performed on SeQuant ZIC HILIC 200 A° column (10 mm × 250 mm, 5 µm), Flow rate: 5.0 mL/min. The elution conditions used are: Buffer A: 5 mmol ammonium acetate (adjust to pH-4 with Acetic Acid), Buffer B: Acetonitrile, Equilibration of the column with 90 % buffer B and elution by a gradient of 90 % to 40 % buffer B during 20 min. Liquid chromatography–mass spectrometry (LCMS) was performed on AP1 2000 LC/MS/MS triple quad (Applied biosystems) with Agilent 1100 series HPLC with G1315 B DAD, using Mercury MS column. All high-resolution mass spectra (HRMS) were recorded on a Waters LCT premier XE mass spectrometer using positive electrospray ionisation (ESI).

Synthesis of NP-01, AUPM-13, AUPM-105, AUPM-209, and AUPM-219 are disclosed in patent numbers WO2011161699, WO2013132317, WO2015033303 as compound numbers 1, 2, 13, 1, and 2 respectively. PM-116 is synthesized using a similar procedure as that of NP-01, with a purity of 98 %. HRMS (ESI) m/z calculated for $C_{11}H_{21}N_4O_7$ $[M+H]^+$: 321.1332, found: 321.1394.

Synthesis of PM-823

Linear synthesis of peptide was carried out on CTC resin using similar procedure as that of NP-01. Peptide was cleaved from the resin as a protected peptide by treating it with 30% of HFIP in DCM for 2 h followed by ether work up to yield 1.6 g of protected peptide. The crude peptide (1.6 g) was used as such for head to tail cyclization using HATU (3 equiv), DIPEA (3 equiv) in THF. The reaction was continued with stirring for 12 hours at room temperature. The progress of reaction was monitored by TLC. The reaction mass was partitioned between ethyl acetate and water.

Organic layer was washed with brine, then organic layer was dried over Na_2SO_4 and evaporated under reduced pressure to give crude. The crude was washed with diethyl ether to yield 1.5g of cyclised peptide. The crude protected cyclised peptide was converted to cyclic peptide by treating with cleavage cocktail, TFA:TIPS:H_2O (9.5:0.25:0.25) at room temperature for 3.0 h. Cleavage mixture was collected by filtration and the resin was washed with DCM. The excess TFA and DCM were evaporated under nitrogen. The crude obtained was washed with diethyl ether followed by lyophilisation yields the crude product. Analytical HPLC was performed using on Luna Omega 5µm PS C_{18} 100A° column (4.6 mm × 250 mm, 5 µm), Flow rate: 1.0 mL / min using Buffer A: 5 m mol ammonium acetate, Buffer B: Acetonitrile. Pure fractions were collected and lyophilized to get 30 mg 97 % pure (HPLC) peptide. HRMS (ESI) m/z calculated for $C_{31}H_{45}N_8O_{14}$ $[M+H]^+$: 753.2977, found: 753.3062

Synthesis of CA-170 (Compound number 4 in patent No. WO2015033299)

Scheme 1

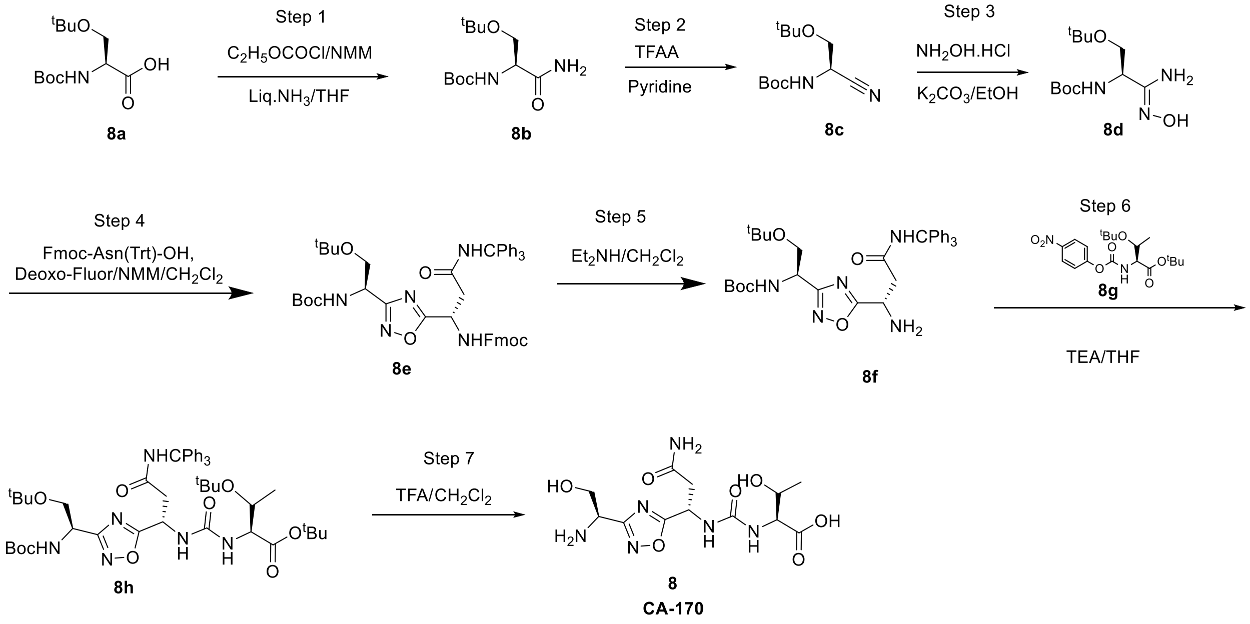

Step 1: Synthesis of tert-butyl (S)-(1-amino-3-(tert-butoxy)-1-oxopropan-2-yl) carbamate

Ethylchloroformate (1.5 g, 13.78 mmol) and N-Methylmorpholine (NMM, 1.4 g, 13.78 mmol) were added to a solution of compound 8a (3 g, 11.48 mmol) in 30 mL of tetrahydrofuran (THF) and stirred at -20 °C. After 20 min. aqueous ammonia (0.77 g, 45.92 mmol) was added to the active mixed anhydride formed in-situ and stirred at 0-5 °C for 20 min. The reaction mixture was evaporated under reduced pressure and partitioned between water and ethyl acetate. Organic layer

was washed with sodium bicarbonate ($NaHCO_3$), citric acid, brine solution, dried over Sodium sulfate (Na_2SO_4) and evaporated under reduced pressure to get 2.9 g of compound 8b (Yield: 96.3%). LCMS m/z calculated for $C_{12}H_{25}N_2O_4$ $[M+H]^+$: 261.1, found: 261.0 $(M+H)^+$.

Step 2: Synthesis of tert-butyl (R)-(2-(tert-butoxy)-1-cyanoethyl) carbamate

Trifluroacetic anhydride (9.7 g, 46.0 mmol) was added to a solution of compound 8b (8 g, 30.7 mmol) in pyridine (24.3 g, 307.0 mmol) and stirred at room temperature for 3 h. The reaction mixture was evaporated under reduced pressure and partitioned between water and ethyl acetate. Organic layer was washed with $NaHCO_3$, citric acid, brine solution, dried over Na_2SO_4 and evaporated under reduced pressure to afford 7 g of compound 8c (Yield: 94.0%). LCMS m/z calculated for $C_8H_{15}N_2O_3$ $[M\text{-}tBu]^+$: 187.2, found 187.2

Step 3: Synthesis of tert-butyl (R,Z)-(1-amino-3-(tert-butoxy)-1-(hydroxyimino)propan-2-yl)carbamate

Hydroxylamine hydrochloride (3 g, 43.37 mmol) and potassium carbonate (6 g, 43.37 mmol) were added to a solution of compound 8c (7 g, 28.91 mmol) in ethanol (70 m L) and stirred at 90 ℃ for 2 h. The reaction mixture was evaporated under reduced pressure and partitioned between water and ethyl acetate. Organic layer was washed with brine solution, dried over Na_2SO_4 and evaporated under reduced pressure. The crude compound was purified by silica gel column chromatography (Eluent: 0-5% ethyl acetate in hexane) to get 4.2 g of compound 8d (Yield: 52.8%). LCMS m/z calculated for $C_{12}H_{26}N_3O_4$ $[M+H]^+$: 276.4, found 276.4

Step 4: Synthesis of tert-butyl ((R)-1-(5-((S)-1-((((9H-fluoren-9-yl)methoxy)carbonyl)amino)-3-oxo-3-(tritylamino)propyl)-1,2,4-oxadiazol-3-yl)-2-(tert-butoxy)ethyl)carbamate

Deoxo-fluor (1.83 g, 8.3 mmol) was added to a solution of Fmoc-Asn(Trt)-OH (4.5 g, 7.5 mmol) in 50 mL of dichloromethane (CH_2Cl_2) and stirred at 0 ℃ for 3 h. The reaction mixture was evaporated to remove dichloromethane and triturated with hexane, decanted and evaporated under vacuum to get the corresponding acid fluoride. NMM (1.17 g, 11.6 mmol) and compound 8d (1.6 g, 5.8 mmol) in THF were added to the acid fluoride and stirred at room temperature for 12 h. Reaction mixture was evaporated to remove THF. Sodium acetate (0.72 g, 8.7 mmol) was added to reaction mass followed by ethanol (50 mL) and stirred at 90 ℃ for 2 h. The reaction mixture was evaporated under reduced pressure and partitioned between water and ethyl acetate. Organic layer was washed with $NaHCO_3$, citric acid, brine solution, dried over Na_2SO_4 and evaporated

under reduced pressure, which was further purified by silica gel column chromatography (Eluent: 0-5% ethyl acetate in hexane) to afford 2.8 g of compound 8e (Yield: 44.4%). LCMS m/z calculated for $C_{50}H_{54}N_5O_7$ $[M+H]^+$: 836.4, found 836.4

Step 5: Synthesis of tert-butyl ((R)-1-(5-((S)-1-amino-3-oxo-3-(tritylamino)propyl)-1,2,4-oxadiazol-3-yl)-2-(tert-butoxy)ethyl)carbamate

To compound 8e (2.3 g, 2.7 mmol) in CH_2Cl_2 (10 mL) diethylamine (10 mL) was added and the reaction mixture was stirred at room temperature for 30 min. The resulting solution was concentrated in vacuum to get gummy residue. The crude compound was purified by neutral alumina column chromatography (Eluent: 0-50% ethyl acetate in hexane then 0-5% methanol in chloroform) to get 1.4 g of 8f (Yield: 90 %). LCMS m/z calculated for $C_{35}H_{43}N_5O_5Na$ $[M+Na]^+$: 636.7, found 636.5

Step 6: Synthesis of tert-butyl N-(((S)-1-(3-((R)-2-(tert-butoxy)-1-((tert-butoxycarbonyl) amino)ethyl)-1,2,4-oxadiazol-5-yl)-3-oxo-3-(tritylamino)propyl)carbamoyl)-O-(tert-butyl)-L-threoninate

The urea linkage was carried out by the coupling compound 8f (2.7 g, 4.39 mmol) in THF (30 mL) at room temperature with compound 8g (1.78 g, 4.39 mmol). The coupling was initiated by the addition of triethyl amine (0.9 g, 8.78 mmol) in THF (10 m L) and the resultant mixture was stirred at room temperature. After completion of 20 h, THF was evaporated from the reaction mass, and partitioned between water and ethyl acetate. Organic layer was washed with water, brine, dried over Na2SO4 and evaporated under reduced pressure to get compound 1h, which was further purified by silica gel column chromatography (Eluent: 0-50% ethyl acetate in hexane) to afford 3.46 g of compound 8h (Yield: 92.10%). LCMS m/z calculated for $C_{48}H_{67}N_6O_9$ $[M+H]^+$: 871.5, found 871.6

Step 7 Synthesis of (((S)-3-amino-1-(3-((R)-1-amino-2-hydroxyethyl)-1,2,4-oxadiazol-5-yl)-3-oxopropyl)carbamoyl)-L-threonine (CA-170)

To a solution of compound 8h (0.22 g, 0.25 mmol) in CH_2Cl_2 (5 m L), trifluoroacetic acid (5 mL) and catalytic amount of triisopropylsilane were added and stirred for 3h at room temperature. The resulting solution was concentrated under reduced pressure to obtain 0.35 g of crude compound. The crude solid material was purified using preparative-HPLC method described under experimental conditions (Yield: 0.027 g, 30%). ^{1}H-NMR (400 MHz, D_2O): δ 1.01 (d, 3H), 2.72 (d, 2H),3.30 (dq, 2H), 3.97 (dd, 1H), 3.99 (t, 1H), 4.05 (q, 1H), 5.25 (q, 1H), 6.41 (d, 1H), 6.99 (s,

1H), 7.13 (d, 1H), 7.49 (s, 1H), ^{13}C-NMR (100.6 MHz, D_2O): δ 20.20, 38.38, 44.24, 50.15, 58.03, 63.68, 66.65, 157.12, 170.39, 170.65, 173.28, 180.35. HRMS (ESI) m/z calculated for $C_{12}H_{21}N_6O_7$ $[M+H]^+$: 361.1393, found: 361.1479

Scheme for Side chain synthesis for step 6

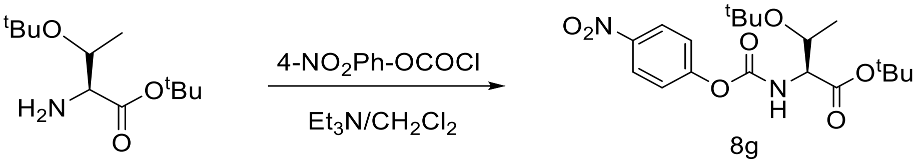

Synthesis of tert-butyl O-(tert-butyl)-N-((4-nitrophenoxy)carbonyl)-L-threoninate (8g):

To the compound H-Thr(tBu)-OtBu (2.1 g, 9.2 mmol) in CH_2Cl_2 (20 mL), triethyl amine (1.49 g, 13.8 mmol) was added and the solution was stirred at room temperature for 5-10 min. To this mixture, solution of 4-Nitrophenyl chloroformate (2.38 g, 11.04 mmol) in CH_2Cl_2 was added and the resultant mixture was stirred at room temperature for 30 min. After completion of reaction, reaction mixture was diluted with CH_2Cl_2 and washed with water and 5.0 M citric acid solution, dried over Na_2SO_4 and evaporated under reduced pressure to get crude compound 8g. The crude was stirred with excess hexane to precipitate out undesired impurities. Filtrate containing active carbamate was concentrated and directly used for step 6.
