# Supplementary material for: PD-1 derived CA-170 is an oral immune checkpoint inhibitor that exhibits preclinical anti-tumor efficacy
Source: Commun Biol. 2021 Jun 8;4:699. doi: 10.1038/s42003-021-02191-1 (PMC8187357; doi:10.1038/s42003-021-02191-1)
Supplement: Supplementary file 3 — Description of Supplementary Files [file 42003_2021_2191_MOESM3_ESM.pdf]

Description of Additional Supplementary Files

File name: Supplementary Data 1

Description: Source data underlying Figure 1-5.
