# Supplementary material for: PD-1 derived CA-170 is an oral immune checkpoint inhibitor that exhibits preclinical anti-tumor efficacy
Source: Commun Biol. 2021 Jun 8;4:699. doi: 10.1038/s42003-021-02191-1 (PMC8187357; doi:10.1038/s42003-021-02191-1)
Supplement: Supplementary file 4 — Reporting Summary [file 42003_2021_2191_MOESM4_ESM.pdf]

nature research

Corresponding author(s): Murali Ramachandra

Last updated by author(s): 29-Apr-2021

Reporting Summary

Nature Research wishes to improve the reproducibility of the work that we publish. This form provides structure for consistency and transparency in reporting. For further information on Nature Research policies, see our Editorial Policies and the Editorial Policy Checklist.

Statistics

For all statistical analyses, confirm that the following items are present in the figure legend, table legend, main text, or Methods section.

n/a	Confirmed	
☐	☒	The exact sample size (*n*) for each experimental group/condition, given as a discrete number and unit of measurement
☐	☒	A statement on whether measurements were taken from distinct samples or whether the same sample was measured repeatedly
☐	☒	The statistical test(s) used AND whether they are one- or two-sided<br>*Only common tests should be described solely by name; describe more complex techniques in the Methods section.*
☒	☐	A description of all covariates tested
☒	☐	A description of any assumptions or corrections, such as tests of normality and adjustment for multiple comparisons
☐	☒	A full description of the statistical parameters including central tendency (e.g. means) or other basic estimates (e.g. regression coefficient) AND variation (e.g. standard deviation) or associated estimates of uncertainty (e.g. confidence intervals)
☐	☒	For null hypothesis testing, the test statistic (e.g. *F*, *t*, *r*) with confidence intervals, effect sizes, degrees of freedom and *P* value noted<br>*Give P values as exact values whenever suitable.*
☒	☐	For Bayesian analysis, information on the choice of priors and Markov chain Monte Carlo settings
☒	☐	For hierarchical and complex designs, identification of the appropriate level for tests and full reporting of outcomes
☒	☐	Estimates of effect sizes (e.g. Cohen's *d*, Pearson's *r*), indicating how they were calculated

Our web collection on statistics for biologists contains articles on many of the points above.

Software and code

Policy information about availability of computer code

Data collection	Flow Cytometry : Beckton Dickenson flow cytometer.<br>ELISA: Biotek ELISA reader or Spectramax ELISA Reader was used to measure the absorbance and delta values generated from the machine were used for analysis and interpretation.<br>LC-MS/MS : Analyst 1.5<br>HSQC NMR : Bruker AVANCEIII spectrometer operating at a 1H resonance frequency of 800 MHz and equipped with a 5 mm cryogenically cooled triple resonance probe
Data analysis	Flow cytometry: Flowjo (Version 6.0) or Cell Quest Pro (Version 6), was used to analyze flow cytometry data.<br>Interpretation of data and dose response curves / percentage proliferation or percentage proliferation rescue were calculated using GraphPad prism (ver 7.0) and MS EXCEL.<br>ELISA: The concentration values were used to interpret the percentage release / rescue using MS EXCEL.<br>LC-MS/MS : Phoenix WinNonlin 8.3<br>HSQC NMR : The data obtained was processed and analysed using TOPSPIN software version 3.5.<br>Animal Studies: One-way ANOVA or Student's t Test was used to compare treatment effect as specified using MS EXCEL/GraphPad Prism

For manuscripts utilizing custom algorithms or software that are central to the research but not yet described in published literature, software must be made available to editors and reviewers. We strongly encourage code deposition in a community repository (e.g. GitHub). See the Nature Research guidelines for submitting code & software for further information.

Data

Policy information about availability of data

All manuscripts must include a data availability statement. This statement should provide the following information, where applicable:

- Accession codes, unique identifiers, or web links for publicly available datasets
- A list of figures that have associated raw data
- A description of any restrictions on data availability

Raw data for all the data reported in the manuscript is available.

Field-specific reporting

Please select the one below that is the best fit for your research. If you are not sure, read the appropriate sections before making your selection.

- [x] Life sciences
- [ ] Behavioural & social sciences
- [ ] Ecological, evolutionary & environmental sciences

For a reference copy of the document with all sections, see nature.com/documents/nr-reporting-summary-flat.pdf

Life sciences study design

All studies must disclose on these points even when the disclosure is negative.

Sample size	Sample size calculations were considered based on previous experience for each experiment and were not predetermined using any statistical analysis. Information regarding the number of replicates in each experiment and the independent experiments for each measurement are disclosed in the manuscript.
Data exclusions	Data were not excluded from analysis
Replication	Reproducibility of data was ensured by using independent experiments or technical replicates. Information regarding the number of replicate in each experiment and the independent experiments for each measurement are disclosed in the manuscript.
Randomization	For in vivo PD evaluation and efficacy studies, animals were randomized and assigned to a treatment group.
Blinding	For the PD evaluation studies, investigators were blinded to the treatment groups.

Reporting for specific materials, systems and methods

We require information from authors about some types of materials, experimental systems and methods used in many studies. Here, indicate whether each material, system or method listed is relevant to your study. If you are not sure if a list item applies to your research, read the appropriate section before selecting a response.

Materials & experimental systems

n/a	Involved in the study	
[ ]	[x]	Antibodies
[ ]	[x]	Eukaryotic cell lines
[x]	[ ]	Palaeontology and archaeology
[ ]	[x]	Animals and other organisms
[ ]	[x]	Human research participants
[x]	[ ]	Clinical data
[x]	[ ]	Dual use research of concern

Methods

n/a	Involved in the study	
[x]	[ ]	ChIP-seq
[ ]	[x]	Flow cytometry
[x]	[ ]	MRI-based neuroimaging

Antibodies

Antibodies used	Details of the commercially available antibodies used in the study are provided in the supplementary files submitted with the manuscript
Validation	Antibodies were validated by the manufacturer according to the standard protocols mentioned in the manufacturer website.

Eukaryotic cell lines

Policy information about cell lines

Cell line source(s)	Jurkat T Cells (E6) - ATCC

	MC38 - NIH<br>CT26 - ATCC<br>B16F10- ATCC
Authentication	Cell lines were procured directly from the vendor. Authentication was performed by the vendor, which includes STR profiling. Additional authentication was not performed.<br>MC38 cell line was used as received from NCI/NIH.
Mycoplasma contamination	Since no unusual growth pattern and/or unresponsive cell lines were observed, mycoplasma testing was not performed.
Commonly misidentified lines (See ICLAC register)	Commonly misidentified cell lines were not used in this study.

Animals and other organisms

Policy information about studies involving animals; ARRIVE guidelines recommended for reporting animal research

Laboratory animals	MC38 Study and B16F10 study: C57Bl/6 (6-8 weeks, male)<br>MC38 Study: SCID/beige (6-8 weeks, male)<br>CT26 Studies: Balb/c (5-7 weeks, male).<br>Pharmacokinetics study: CD-1 (9-11 weeks, male)
Wild animals	This study did not involve the use of wild animals
Field-collected samples	This study did not involve the samples collected from the field.
Ethics oversight	All animal experiments were approved by the Institutional Animal Ethical Committee based on the Committee for the Purpose of Control and Supervision on Experiments on Animals (India) guidelines

Note that full information on the approval of the study protocol must also be provided in the manuscript.

Human research participants

Policy information about studies involving human research participants

Population characteristics	Healthy male or female volunteers in the age group of 18-35 years
Recruitment	Non-smoker, non-alcoholic and without previous immediate medical history of being under medication or exposed to high levels of radiation or hazardous chemicals. No biases involved.
Ethics oversight	Blood collection was initiated after approval from Suraksha Independent Ethics Committee (SIEC), India

Note that full information on the approval of the study protocol must also be provided in the manuscript.

Flow Cytometry

Plots

Confirm that:

- [x] The axis labels state the marker and fluorochrome used (e.g. CD4-FITC).
- [x] The axis scales are clearly visible. Include numbers along axes only for bottom left plot of group (a 'group' is an analysis of identical markers).
- [x] All plots are contour plots with outliers or pseudocolor plots.
- [x] A numerical value for number of cells or percentage (with statistics) is provided.

Methodology

Sample preparation	For cell proliferation assay by CFSE, Human PBMCs or mouse splenocytes were isolated by density gradient centrifugation and cultured in the presence of compounds and recombinant proteins. The cells were harvested after the incubation period and acquired using flow cytometer.<br>For Pharmacodynamic evaluation studies, tumor samples were excised from treated animals, cut into small pieces and incubated with tumor dissociation cocktail for 1 hr at 37 °C in a shaker. The samples were strained and single cell suspension was obtained by centrifugation. The cells were stained with the respective fluorescent tagged antibodies at 4 ° C for 30 mins for surface markers. For intracellular markers, cells were permeabilized in permeabilization buffer overnight and then stained with the respective fluorescent tagged antibodies at 4 ° C for 2 hr.
Instrument	All the flow cytometry experiments were conducted using Beckton Dickenson flow cytometer (three color).
Software	Flowjo (Version 6.0) or Cell Quest Pro (Version 6), was used to analyze flow cytometry data
Cell population abundance	For cell proliferation assay by CFSE, 10,000 cells were acquired from each sample.

For Pharmacodynamic evaluation studies, 20,000-50,000 CD45+ve cells were acquired from each sample. Cell sorting was not performed in any of the experiments.

Gating strategy

Preliminary gating of cells were done on FSC-SSC plot. For CFSE proliferation assay, CFSE stained cells were gated for further acquisition of samples.
For Pharmacodynamic evaluation studies, cells were gated as follows : FSC-SSC- singlets-Live-CD45+ve-CD3+ve - CD4+ve or CD8+ve - Ki67+ve or OX40+ve.
Appropriate single stain controls, isotype controls and FMO (Fluorescence minus one) controls were used to gate the negative and postively stained populations.

☒ Tick this box to confirm that a figure exemplifying the gating strategy is provided in the Supplementary Information.
